# WMAXC: A Weighted Maximum Clique Method for Identifying Condition-Specific Sub-Network

**DOI:** 10.1371/journal.pone.0104993

**Published:** 2014-08-22

**Authors:** Bayarbaatar Amgalan, Hyunju Lee

**Affiliations:** School of Information and Communications, Gwangju Institute of Science and Technology, Gwangju, South Korea; Queen's University Belfast, United Kingdom

## Abstract

Sub-networks can expose complex patterns in an entire bio-molecular network by extracting interactions that depend on temporal or condition-specific contexts. When genes interact with each other during cellular processes, they may form differential co-expression patterns with other genes across different cell states. The identification of condition-specific sub-networks is of great importance in investigating how a living cell adapts to environmental changes. In this work, we propose the weighted MAXimum clique (WMAXC) method to identify a condition-specific sub-network. WMAXC first proposes scoring functions that jointly measure condition-specific changes to both individual genes and gene-gene co-expressions. It then employs a weaker formula of a general maximum clique problem and relates the maximum scored clique of a weighted graph to the optimization of a quadratic objective function under sparsity constraints. We combine a continuous genetic algorithm and a projection procedure to obtain a single optimal sub-network that maximizes the objective function (scoring function) over the standard simplex (sparsity constraints). We applied the WMAXC method to both simulated data and real data sets of ovarian and prostate cancer. Compared with previous methods, WMAXC selected a large fraction of cancer-related genes, which were enriched in cancer-related pathways. The results demonstrated that our method efficiently captured a subset of genes relevant under the investigated condition.

## Introduction

A central problem in network biology is the identification of genes and pathways involved in the same biological processes or physiological conditions. The details of control mechanisms in biological processes can be understood by analyzing interacting neighbors and local patterns. Network structures often have been used to describe these complex bio-molecular pathways and functional modules by representing a whole set of interactions as overlapping sub-networks, each associated with a specific condition [1], [2].

Many methods have been developed to construct bio-molecular networks by comparing multiple sets of microarray data under different conditions. Because expressions of different genes in a series of biological conditions influence each other, correlations between genes have been widely used to analyze microarray gene-expression measurements. Waaijenborg and Zwinderman [3] developed a penalized canonical correlation analysis method to extract a subset of variables that capture the common features among genes by maximizing a canonical correlation between expression of genes. Witten and Tibshirani [4] presented some extension formulas to the sparse canonical correlation analysis as a supervised method, which resulted in the identification of linear combinations of sets of variables that are correlated and associated with its outcome.

To fully understand the complex biological processes, the effective integration of diverse sets of data and knowledge is required. Protein-DNA interaction and gene expression data were combined for regulatory network identification [5]. Integrating protein-protein interaction (PPI) data with gene expression data has also been attempted for the identification of biologically meaningful and cancer-related networks in cancer studies [6]–[9]. The integration increases the accuracy in identifying genes jointly regulated in the same condition. Guo et al. [10] developed an edge-based scoring function for gene-gene co-expression by using both gene expression and PPI data. However, in this method, PPI information is used to define existence of edges in the bio-molecular network so that only gene pairs that are included in the existing PPI network are considered in the scoring function. Lai et al. [11] extended the traditional F-statistic to obtain an expected conditional F-statistic (ECF-statistic), which measures the connectivity between genes. The ECF-statistic was used in a COSINE method [12] to measure gene-gene co-expression from gene expression data. For the use of the PPI information in COSINE, only the number of interactions in a selected sub-network was considered in their scoring function to calculate its sub-network adjust score, instead of using each interaction in the PPI network. A mixed integer linear programming model [13] and an integer linear programming approach [14] were developed to identify differentially expressed pathways using both data sets. However, these two integer linear programming approaches have used expression values of individual genes without accounting for correlation or co-expression information, which might be less informative.

Finding a sub-network that maximizes the score of differential expressions of genes and differential co-expression of gene pairs can be formulated as a combinatorial optimization problem. In practice, bio-molecular networks are often large in scale [15]. Hence, it is impossible to exactly solve a large combinatorial optimization problem within a reasonable time. For examples, in the COSINE method [12], a genetic algorithm was used to find a binary vector, in which 1 or 0 represents presence or absence of a gene in the sub-network, and its space complexity is exponential as 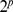, where 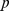 is the total number of genes. In the edge-based method [10], a searching procedure based on simulated annealing was used to find the sub-network. It iteratively tests whether the addition or removal of an edge will increase their sub-network adjust score during the annealing process, and its space complexity is also exponential as 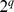, where 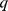 is the number of possible edges (edges in the PPI network). Ideker et al. [6] also used the simulated annealing approach; however, it tended to produce a large sub-network that was often difficult to interpret. Although Rajagopalan and Agarwal [16] and Nacu et al. [17] offered several improvements, they were also based on heuristic techniques with combinatorial selection and required the estimation of additional parameters. Various graph theory-based approaches, such as sequential greedy heuristics [18], [19], have been presented. These heuristics generate a maximal scoring sub-network through the repeated addition of a vertex into a partial sub-network, or by the repeated deletion of a vertex from a sub-network. In addition to heuristics, several local optimization-based approaches have been presented to extract condition specific sub-networks. A method developed by Wang and Xia [15] was inspired by a KKT condition and was used to iteratively find a local minimum from a predetermined initial solution. A large number of local solutions can be found for the non-convex problem. Depending on the selection of the initial solution, many of them might not be significant under the investigated condition, which may give rise to false positives.

**Figure 1:**
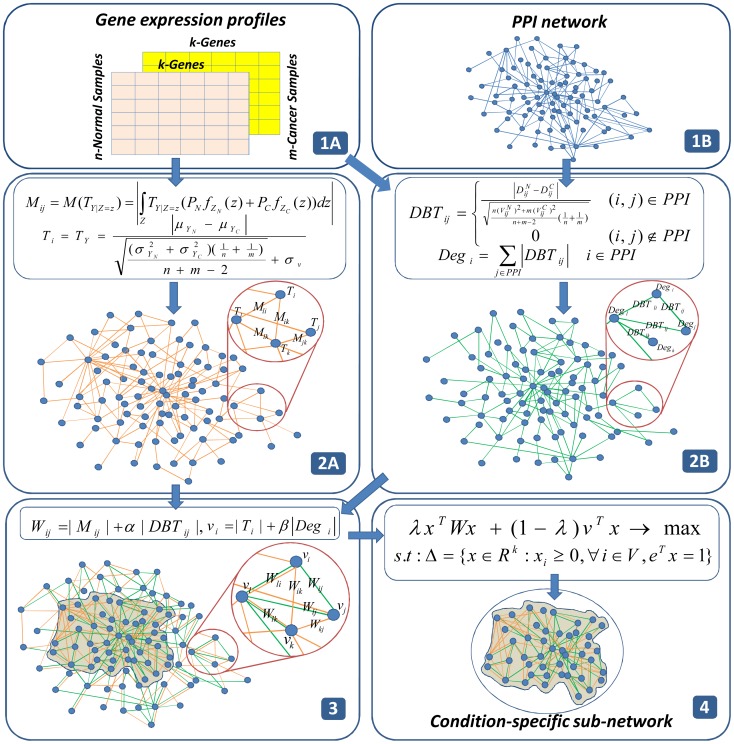
Workflow of WMAXC. (1) Gene expression data consisting of normal and cancer samples (1A) and the PPI network (1B) are used as inputs. (2) We begin by constructing two responsive networks under the investigated condition: In (2A), we use two statistic measurements to construct a bio-molecular network. For each gene, 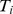 is used to measure activity of gene 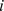 (a node score) and for each pair of genes 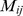 is used to measure connectivity relationship between gene 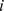 and gene 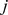 (an edge score). In (2B), for each interaction in PPI network, 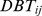 is used to measure activity of interaction behavior between gene 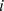 and gene 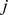 (an edge contribution score from PPI) and for each gene, 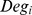 is used to measure the weighted degree of gene 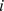 (a node contribution score from PPI) under the condition. (3) We then combine the two responsive networks to construct the background network by assigning node and edge scores to a set of genes. Orange edges represent gene-gene co-expression estimated from only gene expression data and green edges represent activity of interactions in the PPI network. In the process of combining two networks, new edges are included to (2A) although they are not in the existing PPI network. (4) Finally, we solve the constrained optimization problem to obtain the single optimal sub-network.

In our work, we reformulate the sub-network identification problem as a constrained optimization problem for continuous variables. It is an approximation of the general combinatorial problem, based on the theorem posed by Motzkin and Straus [20]. To construct a background network under investigated condition, we used two statistical measurements to represent activity of each genes and interaction behaviors of each pair of genes; a modified *T*-statistic as the differential expression of each gene and a conditional expectation of the modified *T*-statistic as the differential gene-gene co-expression. In our first experiment, we used the two measurements to construct the background network that represents the weight parameters of the optimization problem. We then employed a continuous genetic algorithm, which has the advantage of being capable of jumping out of local solutions, and used a projection procedure that maximizes our objective function under a sparsity constraint. In the second experiment, we reconstructed the background network by integrating PPI information with the gene expression profiles for each gene pair, and obtained a more robust estimation of the neighbors of each gene in the network. We first tested the performance of our method using simulated data sets, and then applied the method to analyze human ovarian and prostate cancer data sets. The results demonstrated that our method efficiently captures relevant interaction behaviors under the investigated conditions. An overview of the workflow is presented in Figure 1, and more detailed descriptions are given in the Methods section.

## Methods

We first describe scoring functions to measure gene expression differences and gene-gene correlations for given two conditions, which generate weight values of nodes and edges in a background network. We then introduce an optimization model to identify the maximal scoring sub-network. Finally, the proposed model is extended to include PPI interactions.

### Scoring function of WMAXC

The entire background network is represented as a graph 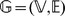, where a set of nodes 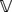 represents genes, and a set of edges 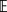 represents the connectivity relationships among these genes. Let 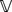 be a set of 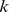 genes. For each gene, 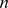 and 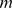 denote the numbers of samples in two different conditions, such as normal and cancer. We then have a gene expression data set, given as two matrices with sizes of 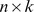 and 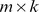. A modified *T*-statistic is used as a scoring function to measure the differential expression of each gene. For each node in 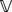, the differential expression value of the corresponding gene 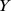 is computed as
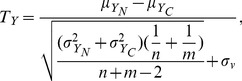
(1)where 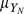, 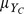 and 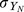, 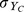 are the sample means and standard deviations of gene 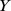 in the normal and cancer conditions, respectively, and 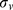 is a constant chosen to minimize the coefficient of variation of *T*-statistic [21] (see more descriptions and Figure S1 in File S1).

For each edge in 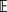, the conditional expectation of the modified *T*-statistic is used to measure differential gene-gene co-expressions across two conditions. To measure the gene-gene correlations for a pair of genes, we assume that samples of genes are jointly normal distributed in a particular condition, such as normal (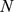) or cancer (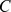). By the assumption, a bivariate normal distribution of two genes 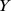 and 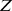 in the normal condition is
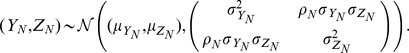



The conditional distribution of 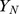 given 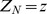 is
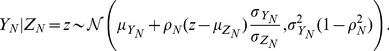
(2)


Similarly, the conditional distribution of 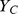 given 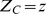 in the cancer condition is
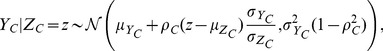
(3)where 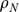 and 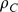 are the sample correlations of a pair of genes 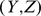 in the normal and cancer conditions, respectively. By replacing mean and variance in Equation (1) with the corresponding conditional means and variances from Equations (2) and (3), we obtain a conditional *T*-statistic as follows.



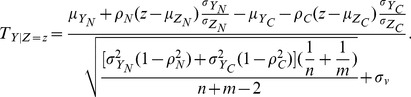
(4)For gene 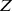, let 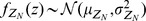 and 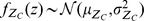 be the probability density function of the normal and cancer conditions, respectively. Then, the probability density function of 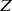 is 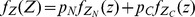, where 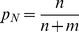 and 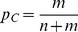 are the probabilities that a sample is selected from the normal or cancer conditions. By calculating the expectation over all samples of gene 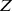, we obtain the connectivity relationship between gene 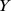 and gene 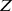 as follows.
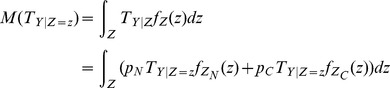
(5)


As we described above, 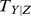, 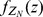 and 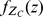 are functions of 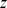 so that the integration in Equation (5) can be numerically computed over all samples in the normal and cancer conditions.

In summary, the modified *T*-statistic is used to measure the differential expression of each gene, and the conditional expectation 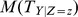 is used to measure differential co-expression of each pair of genes across two conditions. Note that we calculated the differential co-expression patterns 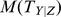 for all pairs of nodes in the background network because the co-expression of two genes might be significant although each gene may not be differentially expressed. In this work, we will use a matrix notation 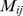 for 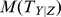 and a vector notation 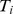 for 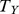, and the matrix 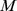 is symmetric, in which its entries represent weights of undirected edges and the entries in the vector 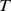 represent weights of nodes in the background network.

### Optimization model

A remarkable connection between the maximum clique problem and a certain standard quadratic programming problem was established [20] by providing an alternative proof of a slightly weaker version of the fundamental theorem [22].

Let 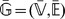 be an unweighted and undirected graph, and 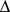 denotes the standard simplex in the *k*-dimensional Euclidean space 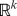, and 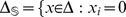 if 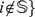 is the face of 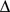 corresponding to a subset 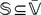. A characteristic vector 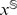 denotes the vector in 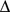 defined by 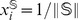 if 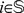 and 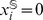, otherwise. The maximum clique problem can then be formulated as the following quadratic programming problem.
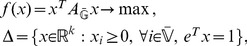
(6)where 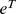 denotes the transpose of the vector 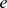 consisting of unit entries, 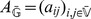 is the adjacency matrix (binary matrix) of 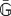, and 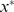 is a global solution of 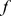 on 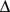. Motzkin et al. [20] proved that the clique number of 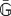 is related to 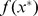 in the following formula.
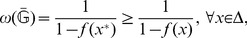
(7)where 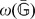 is the size of the maximum clique in 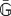. Essentially, they proved that a subset of nodes 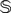 is a maximum clique of 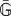 if and only if its characteristic vector 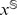 is a global solution of 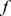 on 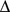 (for a comprehensive review, see [23]).

Based on the theoretical validation, we reformulate the sub-network identification problem as a continuous optimization problem that is an approximation of the general combinatorial problem and a generalization of the problem in Equation (6). The proposed method relates the densest part (a maximum scored clique) of a weighted graph to the optimization of a quadratic function under sparsity constraints. A weaker formula of the maximum clique problem, the optimization problem for identifying a condition-specific sub-network from a bio-molecular network, can be formulated as follows.
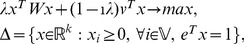
(8)where 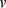 is a vector in which 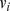 represents a node score measuring differential expression for gene 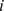, 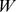 is a symmetric matrix in which 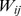 represents an edge score measuring the connectivity strength between gene 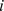 and gene 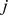, and 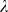 is a positive parameter to balance and to integrate the two terms of the objective function in Equation (8). A *k*-dimensional non-negative vector 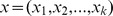, determined by solving our optimization problem, represents the contribution to each gene belonging to the condition specific sub-network. Particularly, 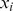 indicates whether its corresponding node is contained in a selected sub-network (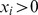) or not (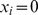). Since we maximize the interconnectivity of sub-network, a gene 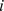 with both a high node confidence score in 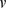 and high confidence scores in 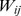 should be selected in the sub-network and its corresponding 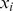 should be assigned to have a high contribution score. Therefore, the subset of variables corresponding to the nonzero elements in the optimal solution 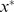 forms the maximum scored sub-network in the background network. Moreover, the genes that have higher contribution scores are more likely to be related to the phenotype (cancer) being analyzed.

### Sub-network identification from gene expression and PPI network data

We extend our model to incorporate the assumption that the significance score of one gene or its interactions in a network depends not only on its own gene expression profile but also on the profiles of its neighbors in the PPI network. Some interactions in the PPI network are activated under the investigated condition while others are not activated. If two genes interact with each other under a particular condition, the expression distance between them might be significantly changed across two conditions (a normal condition and a cancer condition). Based on this assumption, we propose a scoring function called the distance-based *T*-score to measure the change in gene expression distances across two conditions for each pair of genes in the PPI network. This function is used to test the significance score of each interaction in the PPI network.

As described in the section of scoring function of WMAXC, the gene expression profile data are given as two matrices with sizes of 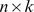 and 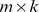. Let 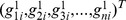 and 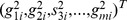 be the samples of gene 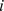 in normal and cancer conditions, respectively, and 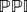 denote a set of pair indexes of genes with interactions in the PPI network. For a pair 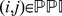, let 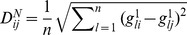 and 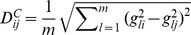 be the normalized distances between gene 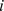 and gene 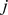 in the normal and cancer conditions, respectively. Then, the distance-based *T*-score, 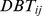, can be formulated as follows.
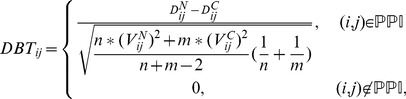
(9)where 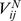 and 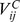 are the geometric averages of the standard deviations of samples of gene 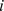 and gene 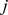 in the normal and cancer conditions, respectively (see more descriptions and Figure S2 in File S1). One of the advantages of using 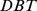 score is that less relevant interactions in the PPI network under the investigated condition can be thinned out. Since both the conditional expectation of the modified *T*-statistic and the distance-based *T*-score are estimated from the same population, we reconstruct our background network by integrating the two types of information. To quantify absolute changes of expression under the investigated condition, we transformed 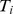, 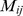, 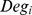 and 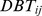 in their absolute values. The weight parameters of the objective function in Equation (8) are then calculated as follows.
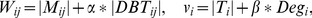
(10)where 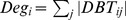 is a weighted degree for gene 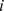 in the PPI network, 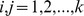 are gene indexes, 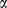 and 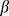 are positive scaling parameters defining the contribution of PPI information to the condition-specific network. We set 
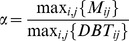
 and 
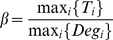
, where 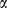 ensures that the maximum of entries in the 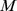 matrix is equal to the maximum of entries in the 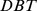 matrix and similar normalization is applied to 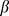.

The PPI network is very sparse; the percentage of interacting protein pairs is only 0.0888% for the ovarian cancer data and 0.1280% for the prostate cancer data. Moreover, some interactions in the existing PPI network might not be significantly activated under the investigated condition. In this case, they might have very small values in 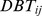 (near to zero). Therefore, the DBT score matrix becomes more sparse, and only the entries corresponding to significant interactions in the PPI network have observable contributions to the matrix 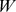 and the vector 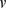. However, the weight matrix 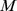 and the weight vector 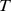 are not sparse, because until now we used all differential expression and co-expression values without considering the significance of them. Therefore, genes with non-significant changes and correlations in expression values contribute their weights to 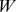 and 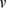. In other words, all non zero entries in 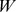 and 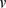 can influence the behavior (slope) of the continuous objective function even though their values are not high. The influence in the objective function may lead the solution to a local maximum. To avoid the local convergence, we define two hard thresholds as follows.
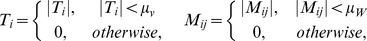
(11)where 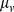 is the mean value of 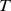, computed as 
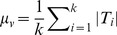
, and 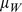 is the maximum of 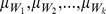, and for each 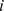, 
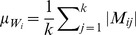
.

### Estimation of weight parameter 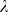


A reasonable balance between the quadratic and linear terms of the objective function in Equation (8) is essential for the stability and robustness of the optimization result. Unfortunately, it is difficult to define an objective criterion for biological relevance. Regarding optimization, the term with greater value should be more informative in our task. To achieve the stability of the solution, we compared the magnitudes of edge and node scores in terms of statistical distributions of score values. Because of the restrictions imposed by the sparsity constraint, the sub-network scores are not affected by their size. For both score terms, sub-network scores of different sizes behave similarly, and the majorities of edge and node score values fall in the region around their means. Therefore, the edge and node score values of a randomly selected sub-network have more chances to fall around the mean (distribution plots are given in Figures S3 and S4 in File S1), which allows the possibility of estimating the magnitudes that make the solution more stable. To estimate the magnitudes of the two terms, we used the following procedure: 1) Randomly sample a large number of sub-networks by selecting points on 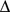. Each point 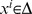 presents a sub-network, and the edge and node scores of the sub-network are calculated as 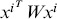 and 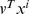, respectively; 2) Compute the means 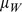 and 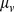, standard deviations 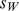 and 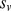 of two score terms; 3) The magnitudes of both edge and node score terms are defined as follows: 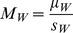, 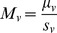; and then 4) We set 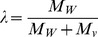.

### Searching for condition specific sub-network

If the matrix 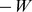 is positive definite, the objective function in Equation (8) is concave (concave maximization is equivalent to convex minimization), and any local solution can also be the global maximum. Unfortunately, in the WMAXC method, the weight matrix 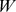 is generally indefinite. There are a large number of local maxima, each representing a densely connected subgraph. Because of the high complexity of the problem, it is a common practice to solve it using metaheuristics, such as evolutionary algorithms. This study implements a combination of a continuous genetic algorithm [24] and a projection procedure [25]–[27] to avoid the local maxima and reduce computational costs.

The continuous genetic algorithm is a parallel search procedure commonly used in a high-dimensional global optimization problem. For constrained optimizations, depending on the shape of the constraint and the dimension of the problem, the implementation of a genetic algorithm should be adapted to a particular problem. Because the standard simplex 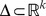 is a subspace of the 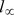 ball 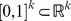 (see Figure S5 in File S1), we first apply the continuous genetic algorithm to maximize the objective function in Equation (8) over 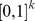 and find a single optimal solution. Let 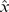 be the global maximum of Equation (8) over 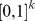. We then project the solution 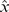 onto 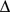 in the Euclidean space 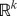 using the algorithm [26] to obtain a sparser solution [27]. Since projection of any point onto convex set is unique (see the projection theorem and Figure S6 in File S1) and the 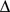 is convex, the problem of finding the Euclidean projection of a vector 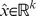 onto 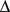 can be described by the following convex optimization problem.
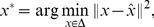
(12)where 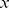 is a *k*-dimensional vector used as an optimization variable, and 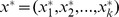 is the projection of 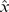 onto 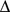 (see File S1). In view of the Lagrangian duality, the optimal solutions of the primal and dual problems are equal to a saddle point. In our task, the optimization variable of the dual problem is simply a scalar. Hence, it is more efficient to solve the dual problem instead of directly solving the primal problem in Equation (12). By considering the well-known result of the projection onto standard simplex [25], the optimal projection 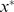 is expressed as follows.
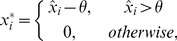
(13)where the dual optimal point 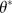 is given by the root of the following equation:



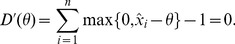
(14)The root 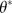 of Equation (14) can be numerically found using the algorithm from [26] and is used in Equation (13). The solution of Equation (13) represents the optimal solution of the problem in Equation (8). After the procedure is completed, the nodes corresponding to non-zero entries in the solution vector are selected to represent the subset of genes forming the condition-specific sub-network.

### Data sets

- Protein-protein interaction data: We used the Human Protein Reference Database released in 2010 [28]. There were 39,240 binary protein-protein interactions involving 9,617 genes. After excluding self-interactions, 37,080 interactions remained.
- Ovarian cancer data: Gene expression data was collected from the TCGA project [29]. It contains 17,814 genes with the expression profiles of 587 cancer samples and 62 normal samples. For the cancer samples, we only considered 332 samples without missing values. For the normal samples, we imputed the missing values in the samples using the Weighted K-Nearest Neighbors method, based on available observations in the samples. For the analysis of ovarian cancer, we considered only the genes that were included in the PPI network, which consists of 8,721 genes and 33,771 interactions.
- Prostate cancer data: Gene expression data was collected from [30] with gene expression omnibus (GEO) accession number GSE3933. It contains the gene expression profiles of 71 cancer samples and 41 normal samples. The gene expression data was initially used in COSINE [12]. In order to compare our results with the results of COSINE using the same data, we used the data set prepared by the authors of COSINE. This data set consists of 5,335 genes with 18,234 interactions in the PPI network.

## Results

### Simulation studies

We showed the performance of WMAXC on simulated data by comparing it with COSINE [12], because COSINE was initially compared to several other methods, including jActiveModules [6], an edge-based method [10], and a local method [15]. We constructed five simulation data sets from multivariate normal distributions: four case data sets and one reference data set.

Each data set consisted of 1,000 variables (genes) for 50 samples. For each gene 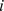, we draw the mean 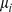 and the standard deviation 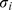 from the uniform distribution on the observed range of normal data [−0.5,0.5], and then estimated the correlation coefficient 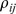 for each pair of genes (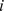,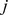). Let 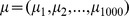 denote the mean vector, and 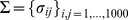 denote the covariance matrix, where 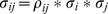 is the covariance between gene 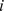 and gene 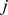.

We first simulated the reference data from joint normal distribution 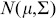, and the reference data was compared to each of the four case data sets. In each of four case data sets, 200 of 1,000 genes were selected as significant genes. For the particular genes, some shifts in the mean expressions and higher correlations among the genes were assigned to represent significant alterations in expression values. Let 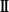 denote the set of indexes for the significant genes.
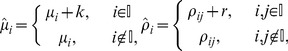
(15)


In each of four case data sets, the expression data was simulated from jointly normal distribution 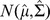, where 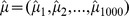 is the mean vector, and 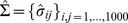 is the covariance matrix and its entries were calculated as 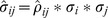. For case data 1, 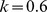 and 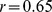 were used in Equation (15), 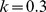 and 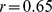 for case data 2, and 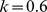 and 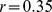 for case data 3, and 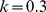 and 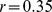 for case data 4. Compared to the results of COSINE, WMAXC provided higher accuracy in sub-network size, recall, precision, and F-measure (Table 1). In the simulated data, the sub-network sizes identified by COSINE were smaller than desired, whereas WMAXC identified approximately 200 genes. Regarding the running time, WMAXC was much faster than COSINE; in the analysis on simulated data, it took 10 minutes to convergence, while COSINE took around 30 hours.

**Table 1:** Comparison on simulated data with COSINE.

methods	WMAXC				COSINE			
Case data	Case 1	Case 2	Case 3	Case 4	Case 1	Case 2	Case 3	Case 4
sub-network size	204	230	219	237	137	178	126	128
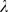	0.157	0.215	0.087	0.223	0.61	0.85	0.11	0.39
Recall	1	0.97	1	0.975	0.66	0.685	0.61	0.605
Precision	0.9803	0.8434	0.9132	0.8227	0.9635	0.7696	0.9682	0.9453
F-measure	0.9901	0.9023	0.9546	0.8924	0.7833	0.7248	0.7484	0.7378

Recall, precision and F-measure are defined as follows: Recall (R) 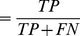, Precision (P) 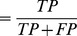 and F-measure 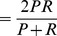, where TP, FP, and FN represent true positive, false positive, and false negative, respectively.

### Comparison with other methods on real data sets

We applied the WMAXC method on gene expression profiles of two real data sets to identify a cancer type specific sub-network. Then, the performance of WMAXC was compared to other methods, COSINE [12] and BMRF [5].

#### Comparison on ovarian cancer

To evaluate whether the genes in the identified sub-network are related to ovarian cancer, we used a set of 379 experimentally verified ovarian cancer-related genes from the Dragon Database of Genes [31] as reference genes. Among the 379 genes, 315 were included in the 8,721 genes of our data set. For WMAXC, the initialization parameters of the genetic algorithm were set as follows: the number of iterations  = 60,000, mutation rate  = 1/(k+1) and crossover rate  = 0.5, where k is the number of optimization variables (the number of genes). We first applied our method using only the gene expression profile data, and then integrated the PPI network with gene expression profiles using the Distance Based *T*-score as described in the Methods section. Performances of the two approaches are shown in Table 2. The fold enrichment of the genes selected among the ovarian cancer-related genes had increased from 1.828 to 2.454 when the PPI network was integrated with the gene expressions, compared to using only the gene expressions. A list of 100 genes with the highest contribution scores to the condition-specific network is given in Table S1 in File S1. Using a CPU with 3.40 GHz and 32 GB RAM, it took 26 hours to search for the maximal scoring sub-network. To test whether the genetic algorithm reached the convergence, we quantified the variations in objective function values for the population in each iteration. For both ovarian and prostate cancer data, the variation was almost zero after running 60,000 iterations, and the minimum values of the objective function became stable in the last 10,000 iterations. This result suggests that the solution had reached convergence.

**Table 2:** Performance on ovarian cancer data.

Methods	COSINE	BMRF	WMAXC1	WMAXC2
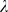	0.871	-	0.173	0.2715
Selected genes	806	916	567	643
Recovered interactions	275	635	483	2015
Recovered genes	36	58	38	57
Fold enrichment	1.237	1.753	1.828	2.454

WMAXC1 represents the results obtained using only gene expression profile data, whereas the WMAXC2 results were obtained by integrating gene expression profiles and PPI network data. ‘Fold enrichment’ was used to evaluate the performance of the methods and was calculated as 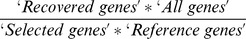, where ‘Selected genes’ is the number of selected genes by the method, ‘Reference genes’ is the number of reference genes from the Ovarian Cancer Dragon Database of genes, ‘Recovered genes’ is recovered genes by the method among the reference genes, and ‘All genes’ represents all genes in the entire network. ‘Recovered interactions’ represents the number of interactions recovered from the PPI network.

For the COSINE method, both gene expression profile and PPI data were used. After five different 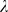
's were tried, the 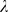 giving the highest adjusted score of the scoring function was used. As a result, the sub-network with a size of 806 was selected. The fold enrichment was 1.237, and it took around 56 hours with 1,000 iterations. For the BMRF method, both gene expression profile and PPI data were used. In addition, a set of hub genes related to the investigated condition was required as an input. Hence, we collected a set of 209 hub genes from KEGG, consisting of genes included in the ovarian cancer-related pathways, such as ubiquitin, coagulation, and hedgehog signaling pathways. BMRF extracted the sub-network of the size of 916 genes, and the fold enrichment was around 1.75. However, its accuracy was depending on the choice of the set of hub genes; for a set of randomly selected genes, the fold enrichment was decreased to 1.041. Overall, the comparison demonstrated that WMAXC outperformed both the COSINE and BMRF methods on the real data set.

#### Comparison on prostate cancer

For the method evaluation, 703 genes related to prostate cancer from the Dragon Database of Genes [32] were used as reference genes. Among the 703 genes, 400 were included in our dataset. 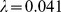 was used for the WMAXC method, and the initialization parameters of the genetic algorithm were the same as those used in the analysis of the ovarian cancer data. WMAXC extracted a sub-network of the size 539 and a fold enrichment of 2.35. A list of 100 genes with the highest contribution scores to the condition specific network is given in Table S2 in File S1. COSINE selected a relatively smaller network with the size of 243 and a fold enrichment of 1.262 [12]. With a set of hub genes included in the mark signaling pathway from KEGG, BMRF selected the sub-network with the size of 601 genes and a fold enrichment of 2.086. However, for a set of randomly selected genes, the fold enrichment was decreased to 0.98. Performances of methods are summarized in Table S3 in File S1, confirming that WMAXC outperformed the other two methods.

### Analysis on real data

**Figure 2:**
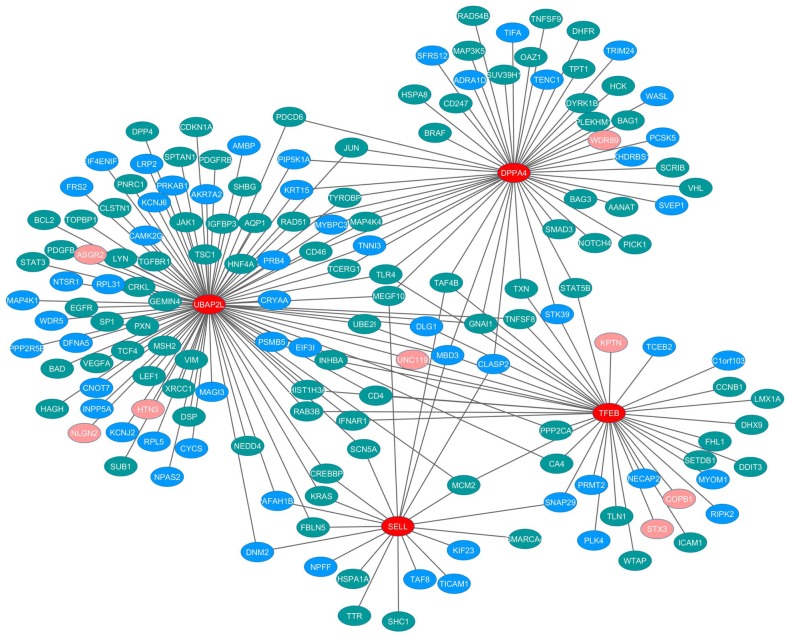
The four candidate genes for ovarian cancer and their neighbor genes in the condition specific network. The four candidate ovarian cancer-related genes are colored in red, ovarian cancer-related genes in green, cancer-related genes in blue and the remaining genes in pink. Edges represent significant co-expressions between genes in the given ovarian cancer.

Although only 57 out of 643 genes forming the condition-specific sub-network were included in DDOC as shown in Table 2, some ovarian cancer-related genes might not be included in DDOC. Hence, we manually checked whether 20 candidate genes with the highest contribution scores were ovarian cancer-related genes. Among them, 16 genes were known to be related to ovarian cancer by DDOC or the manual literature search (Table S4 in File S1). The remaining four genes are SELL, UBAP2L, TFEB and DPPA4. Although there were no evidences of their involvements in ovarian cancer development, these four genes were highly co-expressed with other ovarian cancer and cancer related genes in the condition specific network. As shown in Figure 2, they directly shared significant co-expression patterns with 159 neighbors. Surprisingly, 59.1% (94/159) of neighbors of these four genes are ovarian cancer-related genes and 35.8% (57/159) are other cancer-related genes. A list of these genes with their literature evidences is shown in Table S5 in File S1. For only 5% (8/159) of neighbor genes, we cannot find literature evidences showing their relevance to cancer. We further investigated that these four genes were actively involved in the other cancer types and biological phenomenon. L-selectin, SELL, is a member of a family of adhesion receptors that play important roles in lymphocyte-endothelial cell interactions. Resto et al. [33] investigated adhesive interactions between lymphocytes and head and neck cancer cells (HNSCC cells) under shear stress, and the interactions can be mediated by L-selectin. Kuiper et al. [34] investigated that upregulation of the transcription factor TFEB in some particular chromosomal position may play an important role in the regulation of renal cancer progression. Maldonado-Saldivia et al. [35] provided an evidence that DPPA4 is downregulated during fetal germ line progression and this process might be required to facilitate appropriate germ line differentiation. Moreover, it may provide an implication in the development of germ cell cancer in human. Although there was no much evidence of their involvements in ovarian cancer, our results suggest that these genes might be closely related to ovarian cancer progression.

By applying the WMAXC method to the ovarian cancer data, we identified a biologically meaningful sub-network involved in many ovarian cancer related pathways. Measured using Database for Annotation, Visualization and Integrated Discovery (DAVID) [36], 60 pathways were significantly enriched by the KEGG pathway, including the ErbB signaling pathway, the Notch signaling pathway, and the TGF-*β* signaling pathway (Table S6 in File S1). The epidermal growth factor receptor (EGFR) is a member of the ErbB family of tyrosine kinase receptors. Overexpression of EGFR and its downstream targets are associated with resistance to chemotherapy for ovarian cancer [37]. Ovarian cancer cells, in which Notch3 was frequently amplified and overexpressed, are dependent on the Notch3 signaling pathway for cellular survival and growth. Notch3 expression is also associated with chemo resistance in ovarian high-grade serous carcinoma [38]. The TGF-*β* signaling pathway is activated in ovarian cancer, and the inhibition of this pathway by a small molecule is a promising strategy in the treatment of ovarian cancer [39].

The sub-network identified using the prostate cancer data included 62 significantly enriched KEGG pathways, including prostate cancer, neurotrophinn signaling, MAPK signaling, Wnt signaling, TGF-*β* signaling, chemokine signaling pathways, and the regulation of actin cytoskeleton (Table S7 in File S1). For instance, it has been demonstrated that the progression of prostate cancer is affected by changes in the expression of auctocrine neurotrophins [40]. MAPK signaling is shown to be activated in prostate cancer, especially in later stages of the disease [41]–[43], and it was recently suggested that the MAPK signaling pathway may be a target for prostate cancer therapy, if it is inhibited simultaneously with other pathways, such as PI3K/AKT signaling [44]. The upregulation of some Wnt pathway members was observed in ERG-positive prostate cancers, and it has been shown that knockdown of the ERG gene in VCaP prostate cancer cells causes an activation of cell adhesion and expression changes in Wnt signaling. These findings were validated by gene expression data from both clinical prostate cancer samples and from ERG over-expressing non-transformed prostate epithelial cells [45]. Several studies have shown that changes in the levels of TGF-*β* pathway components are related to prostate cancer progression and cellular responses [46], [47], and [48]. In addition, chemokine signaling pathways and the regulation of actin cytoskeleton have been studied and experimentally validated to be associated with prostate cancer [15], [49].

In summary, the analysis of both simulated and real data provides evidence that the WMAXC method can yield new insights that contribute to a better understanding of diseases.

## Discussion

Our main goal was to design an algorithm that reveals a subset of genes closely related to a particular disease. Based on an optimization framework, we proposed an effective method, WMAXC, for identifying a condition-specific sub-network under a particular condition. WMAXC has the following advantages: (1) It extracts the global optimal sub-network that exhibits significant alterations across two phenotypes; WMAXC considers the weighted contributions of both expression difference for each single gene and the differential correlation of each pair of genes. (2) WMAXC effectively integrates diverse sets of data and knowledge to construct the background network under a particular condition. (3) An optimization formulation with strong theoretical validation is used to represent a continuous version of the general combinatorial problem for identifying a condition-specific sub-network. (4) WMAXC considers all nodes and edges at the same time to search a single optimal sub-network. (5) Genetic algorithm and a projection procedure are combined to approximate the global solution to our problem. (6) A weight parameter 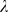 is chosen to make the solution stable to the problem, and it is also adaptive to the specific dataset being analyzed.

WMAXC integrates gene expressions and the PPI network, and the positive scaling parameters 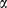 and 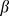 in Equation (10) are contribution factors of the PPI network in identifying disease-specific genes. We expect that for larger 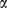 and 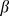 values, the accuracy will increase with a high-quality PPI network, while false positive genes might be included with a low-quality PPI network. In the Results section, we used a high-quality PPI network, and 
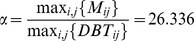
 and 
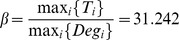
 provide a stable performance, and the fold enrichment is relatively high as around 2.45. To show how the performances of the method are affected depending on PPI data quality and scaling parameters, we simulated three PPI data sets with different qualities by randomly removing a fraction of existing edges from the PPI network and randomly adding the same numbers of edges into the PPI network. Then, the simulated PPI data sets were used with three different sets of scaling parameters in our model and corresponding results for each scaling parameter are given in Table 3. For relatively high-quality data sets such as the original PPI and data set-1, the fold enrichment was increased with larger values of 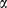 and 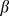. On the other hand, for the low-quality PPI data sets such as data set-3, the fold enrichment was decreased with larger values of 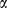 and 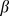.

**Table 3:** Performances on simulated PPI data with different scaling parameters.

Parameters	Original PPI network	PPI data set-1	PPI data set-2	PPI data set-3
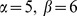	2.2704	1.7681	2.0443	1.7569
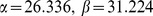	2.454	2.3092	2.2176	1.615
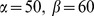	2.4592	2.3843	2.1657	1.5638

For the PPI data set-1, set-2 and set-3, 30%, 50% and 70% of edges from the original data are randomly removed and then the same number of edges are randomly added, respectively. Performances are measured using the fold enrichment, which is described in Table 2.

WMAXC is flexible. It can be simply adapted to directed graphs or even to the integration of gene expression and pathways. For example, instead of using a PPI network, the union of regulatory pathways can be used to represent directed interaction and to compute the DBT score for a pair of genes. In this case, the weight matrix 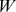 is non-symmetric, and only slight modifications are required to construct a bio-molecular network from the gene expression profile. The solution to the constrained optimization problem can be approximated by combining the genetic algorithm and the projection procedure.

## Supporting Information

File S1 **Supplementary material.** The combined supporting information file contains multiple supporting Figures, Tables and Descriptions of some fundamental concepts used in the work.
(DOCX)
