# Supplementary material for: WMAXC: A Weighted Maximum Clique Method for Identifying Condition-Specific Sub-Network
Source: PLoS One. 2014 Aug 22;9(8):e104993. doi: 10.1371/journal.pone.0104993 (PMC4141761; doi:10.1371/journal.pone.0104993)
Supplement: File S1 — Supplementary material. The combined supporting information file contains multiple supporting Figures, Tables and Descriptions of some fundamental concepts used in the work. (DOCX) [file pone.0104993.s001.docx]

**WMAXC: a weighted maximum clique method for identifying condition-specific sub-network**

Bayarbaatar Amgalan and Hyunju Lee

School of Information and Communications, Gwangju Institute of Science and Technology, Gwangju, Republic of Korea

**Minimization of the coefficient of variation** $\frac{\boldsymbol{\sigma}}{\boldsymbol{\mu}}$

The coefficient of variation is defined as the ratio of the standard deviation $\sigma$ to the mean $\mu$ which is inverse of the signal to noise ratio. As we described, the differential expression for each gene is calculated as $T_{i}(c)=\frac{\mu_{N_{i}}-\mu_{C_{i}}}{\sqrt{\frac{\left( \sigma_{N_{i}}^{2}+\sigma_{C_{i}}^{2} \right)(\frac{1}{n}+\frac{1}{m})}{n+m-2}}+C}$, where $\mu_{N_{i}},\mu_{C_{i}}$ and $\sigma_{N_{i}},\sigma_{C_{i}}$ are the sample means and standard deviations of gene $i$ in the normal and cancer conditions, respectively,$i=1,2,\ldots,k$.

To compare values of $T_{i}(c)$ over all genes, the distribution of $T_{i}(c)$ should be independent of the gene expression level. At low expression levels, variance of $T_{i}(c)$ can be high because of small values of $\sqrt{\frac{\left( \sigma_{N_{i}}^{2}+\sigma_{C_{i}}^{2} \right)(\frac{1}{n}+\frac{1}{m})}{n+m-2}}$. To ensure that the variance of $T_{i}(c)$ is independent of gene expression, a small positive constant $c$ is added to the dominator of $T_{i}(c)$.

Suppose that $\mu_{T}(c)$ and $\sigma_{T}(c)$ are the mean and standard deviations of $T_{i}(c)$ over all indexes $i$. Then, we describe a function of $c$ as follows: $f\left( c \right)=\frac{\sigma_{T}(c)}{\mu_{T}(c)}$.

The value of $\sigma_{v}$ is chosen to minimize the coefficient of variation $f\left( c \right)$. For our simulated case data 1 versus reference data, $\sigma_{v}=0.084$ is the value corresponding to the minimal coefficient of variation (see Figure S1).

**Distance-based T-score**

In this work, we construct the background network under a particular condition by integrating gene expression profile data with the PPI network. Although currently available PPI interaction data does not provide condition specific information, a part of the interactions among a set of genes (proteins) might be activated under a particular condition. Therefore, it might be inadequate to directly take all interactions as the edges of the network; less relevant interactions should be thinned out under the investigated condition and new interaction behaviors should be collected under the condition. To address this issue, we propose a scoring function to measure the connectivity strength of each interaction in the PPI network under the particular condition. We assume that if two genes interact with each other under a particular condition, the distance between them should be significantly changed across two groups (see Figure S2).

**Estimation for weight parameter** $\boldsymbol{\lambda}$

The reasonability of the proposed $\lambda$ estimation procedure is based on the assumption that the term with greater score value is more significant and informative than the other one. For example, in the analysis of the ovarian cancer data, the weight parameter is estimated as $\lambda$=0.2715, which means that the node term is more informative than the edge term, therefore it should have more weight. In Figure S3,for randomly sampled 10,000 sub-networks from ovarian cancer data, the distribution of positive edge score is presented in left-hand side and the distribution of edge score term in the objective function is presented in right-hand side. In Figure S4, for the randomly sampled 10,000 sub-networks from prostate cancer data, the distribution of positive node score is presented in left-hand side and the distribution of node score term in the objective function is presented in right-hand side.

**Optimal projection onto the standard simplex**

In the first-orthant, $l_{1}$ norm (not $l_{1}$ norm ball) is called standard simplex. This type of constraints leads to sparse solutions (Tibshirani, 1996). For many applications of high-dimensional global optimization problems, obtaining a sparse solution which clearly interprets results is crucial. One way to reach a sparse solution is to find the projection of a solution onto a affine subspace, such as $l_{1},l_{2},l_{\infty}$ norms and $\Delta$ standard simplex (Kyrillidis,2013). (See Figure S5)

The problem of finding the Euclidean projection of a vector $\hat{x}\in R^{k}$ onto the standard simplex can be formulated as following convex optimization problem:

$$x^{*}=arg\max_{x\in\Delta} \left\| x-\hat{x} \right\|^{2}$$

where $\Delta$ is the standard simplex set: $\Delta=\{x\in R^{k}:x_{i}\geq0, \forall i\in V,e^{T}x=1\}$ and the optimal projection $k$-dimensional non-negative vector $x^{*}$ represents the condition specific sub-network. It is a subset of nodes corresponding to the nonzero elements in the optimal solution $x^{*}$ forming a maximum scored clique in graph $G$ (condition specific sub-network in the background network). In addition, after projection is done, many entries of a vector$x$ corresponding to less significant genes become 0 since $l_{1}$ norm is more sparse.

Various convex optimization based methods exist for finding the projection a point onto the standard simplex. For instance, Michelot (1986) presented an efficient algorithm with a theoretical proof of optimality based on the Lagrangian condition. Similarly, a dual optimality condition was used to find the projection in the algorithm of Songsiri (2011). Both algorithms were shown to be efficient for finding projection of a point onto standard simplex (For comprehensive review, see following theorem and its proof).

**Projection theorem**: Let $C$ be a nonempty closed convex set.

1. For any $\hat{x}\in R^{n}$, there exists a unique vector

$$x^{*}=P_{C}\left( \hat{x} \right)=arg\min_{x\in C} \left\| x-\hat{x} \right\|$$

called the projection of $\hat{x}$ on $C$.(Figure S6)

2. The $P_{C}(\hat{x})$ could be defined as the only vector with the property

$$\left( y-P_{C}\left( \hat{x} \right) \right)^{T}\left( \hat{x}-P_{C}\left( y \right) \right)\leq0, \forall y\in C$$

If the $C$ is affine and $S$ is a subspace parallel to $C$ then the above can be replaced with

$$(\hat{x}-P_{C}(\hat{x}))\in S^{\perp}$$

3. The function $P_{C}(\hat{x})$ is continuous and non-expansive:

$$\left\| P_{C}\left( \hat{x} \right)-P_{C}(y) \right\|\leq\left\| \hat{x}-y \right\|$$

4. The distance function

$$d\left( \hat{x},C \right)=\min_{x\in C} \left\| \hat{x}-x \right\|$$

is convex.

**Proof**. (1) Follows from the theorem (Weierstrass theorem).

(2) We use notation $x^{*}=P_{C}(\hat{x})$. Clearly, $x^{*}$ has to lie on the boundary of $C$. Also, the $x^{*}$ has to satisfy the condition

$$\frac{\partial}{\partial\varepsilon}\left\| x^{*}+\varepsilon x-\hat{x} \right\|\geq0$$

where the $x$ is taken among all directions such that $x^{*}+\varepsilon x$ remain in $C$ for small $\varepsilon>0$. The differentiation reveals that

$$\left\langle x,x^{*}-\hat{x} \right\rangle\geq0.$$

For any $y\in C,$ the difference $y-x^{*}$ is a valid $x$. Hence, the (2) follows.

(3) Since $P_{C}(\hat{x})\in C$ we can write from (2)

$$\left\langle P_{C}\left( y \right)-P_{C}\left( \hat{x} \right),\hat{x}-P_{C}\left( \hat{x} \right) \right\rangle\leq0,$$

$$\left\langle P_{C}\left( \hat{x} \right)-P_{C}\left( y \right),y-P_{C}\left( y \right) \right\rangle\leq0.$$

We add the above two inequalities and obtain

$$2\left\langle P_{C}\left( y \right)-P_{C}\left( \hat{x} \right),\hat{x}-P_{C}(y)-P_{C}\left( \hat{x} \right) \right\rangle\leq0.$$

Hence,

$$\left\langle P_{C}\left( \hat{x} \right)-P_{C}\left( y \right) \right\rangle^{2}\leq\left\langle P_{C}\left( \hat{x} \right)-P_{C}\left( y \right),\hat{x}-y \right\rangle$$

$$\leq\left\| \left\langle P_{C}\left( \hat{x} \right)-P_{C}\left( y \right) \right\rangle\right\|\left\| \hat{x}-y \right\|$$

(4) follows from (3) and definition of convexity.

**Reference**

1. Chen,L. et al. (2012) Identifying protein interaction subnetworks by a bagging Markov random field- based method. Nucleic Acids Res, 42, e42.

2. Chen,Y. and Ye,X. (2011) Projection Onto A Simplex. Cornell University Library, arXiv:1101.6081.

Dennis,G. et al. (2005) DAVID: database for annotation, visualization, and integrated discovery. Genome Biol, 4, R60.

3. J. Duchi, S. et al. (2008) “Efficient projections onto the l 1-ball for learning in high dimensions,” in *Proceedings of the 25th international conference on Machine learning*. ACM, 2008, pp. 272–279.

4. Kanehisa,M. and Goto,S. (2000) KEGG: kyoto encyclopegia of genes and genomes. Nucleic Acids Res, 28, 27-30.

5. Kyrillidis,A. et al. (2013) Sparse projections onto the simplex. 30th International Conference on Machine Learning (ICML), 28(2), 235-243.

6. Ma,H. et al. (2011) COSINE: COndition-SpecIfic sub-NEtwork identification using a global optimization method. Bioinformatics, 27, 1290-1298.

7. Michelot,C. (1986) A finite algorithm for finding the projection of a point onto the canonical simplex of $R^{n}$. J. Optim. Theory Appl, 50(1), 195-200.

8. Songsiri,J. (2011) Projection onto an l1-norm Ball with Application to Identification of Sparse Autoregressive Models. Asean Symposium on Automatic Control, Vietnam.

9. Tibshirani,R. (1996) Regression Shrinkage and Selection via the Lasso. Journal of the Royal Statistical Society. Series B, 58, 265-288.

10. Witten, D.M. and Tibshirani, R. (2007) A comparison of fold-change and the t-statistic for microarray data analysis. Stanford University, 1-13.

11. Kaur, M. et al. (2008) Ddpc: Database for exploration of functional context of genes implicated in ovarian cancer. Nucleic Acids Research 37, D820-D823.

12. Resto, V.A. et al (2008). L-selectin-mediated Lymphocyte-Cancer Cell Interactions under Low Fluid Shear Conditions. The Journal of Biological Chemistry 283, 15816-15824.

13. Yang, X. et al. (2006) Akt-Mediated Cisplatin Resistance in Ovarian Cancer: Modulation of p53 Action on Caspase-Dependent Mitochondrial Death Pathway. Cancer Research 66, 3126-3136.

14. Kupryjanczyk, J. et al (2013) **Ovarian small cell carcinoma of hypercalcemic type – evidence of germline origin and smarca4 gene inactivation.** POL J PATHOL 64 (4), 238-246.

15. Muratovska, A. et al (2003)Paired-Box genes are frequently expressed in cancer and often required for cancer cell survival. Oncogene 22, 7989–7997.

16. Naz R, and Dhandapani L (2010) Identification of human sperm proteins that interact with humanzonapellucida3 (zp3) using yeast two-hybrid system. Journal of Reproductive Immunology 84, 24-31.

17. Kuiper R, et al. (2003) Upregulation of the transcription factor tfeb in t(6;11)(p21;q13)-positive renal cellcarcinomas due to promoter substitution. Human Molecular Genetics 12, 1661-1669.

# 18. Terauchi M, et al. (2007) Possible involvement of TWIST in enhanced peritoneal metastasis of epithelial ovarian carcinoma. Clinical & Experimental Metastasis 24(5), 329-339

19. Katoh, M. (2007) Integrative genomic analyses on HES/HEY family: Notch-independent HES1, HES3transcription in undifferentiated ES cells, and Notch-dependent HES1, HES5, HEY1, HEY2, HEYL transcription in fetal tissues, adult tissues, or cancer. International Journal of Oncology, 461-466.

20. Jinawath, N. (2010) Oncoproteomic analysis reveals co-upregulation of RELA and STAT5 in carboplatin resistant ovarian carcinoma. Plos One 5(6):e11198.

21. Malonye, A et al (2007) Gene and Protein Expression Profiling of Human Ovarian Cancer Cells Treated with the Heat Shock Protein 90 Inhibitor 17-Allylamino-17-Demethoxygeldanamycin, Cancer Research 67, 3239-3253.

22. Wiedlocha, A et al (2005) Phosphorylation-regulated nucleo cytoplasmic trafficking of internalized fibroblast growth factor-1. Molecular biology of the cell 16(2), 794-810.

23. Zhang,J-H et al (2012) The *EIF4EBP3* translational repressor is a marker of *CDC73* tumor suppressor haplo insufficiency in a parathyroid cancer syndrome. Cell Death & Disease 3(3), 266.

24. Gorringe, K.L. et al (2007) High-resolution single nucleotide polymorphism array analysis of epithelial ovarian cancer reveals numerous micro deletions and amplifications. Clinical Cancer Research 13, 4731-4739.

25. Lee, J-Y. et al (2012) Chicken Pleiotrophin: Regulation of Tissue Specific Expression by Estrogen in the Oviduct and Distinct Expression Pattern in the Ovarian Carcinomas. Plos one 10.1371/journal.pone.0034215.

26. Maldonado-Saldivia J, et al. (2007) Dppa2 and dppa4 are closely linked sap motif genes restricted to pluripotent cells and the germ line. Stem Cells 25: 19-28.

**Supplementary Figures**

**
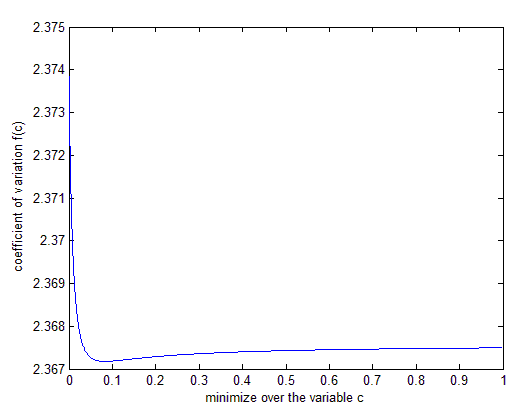
**

**Figure S1:** The coefficient of variation is defined as the ratio of the standard deviation $\sigma$ to the mean $\mu$ which is inverse of the signal to noise ratio: Suppose that $\mu_{T}(c)$ and $\sigma_{T}(c)$ are the mean and standard deviations of $T$ statistic over all indexes $i$. Then, we describe a function of $c$ as follows: $f\left( c \right)=\frac{\sigma_{T}(c)}{\mu_{T}(c)}$.

The value of $\sigma_{v}$ is chosen to minimize the coefficient of variation $f\left( c \right)$. For our simulated case data 1 versus reference data, $\sigma_{v}=0.084$ is the value corresponding to the minimal coefficient of variations.

**Figure S2:** We assume that if two genes interact with each other under a particular condition, the distance between them should be significantly changed across two groups. The DBT-score matrix describes weighted contribution of each interaction in the PPI network to background network.

Edge score term of randomly selected sub-networks

Positive scores for edges

Frequency

Frequency

**Figure S3:** For the randomly sampled 10,000 sub-networks from the ovarian cancer data, the distribution of absolute edge scores(expectation of the T-statistic) is presented in the left-hand side and the distribution of edge score terms in the objective functions is presented in the right-hand side.

Frequency

Positive scores for nodes

Node score term of randomly selected sub-networks

Frequency

**Figure S4:** For the randomly sampled 10,000 sub-networks from the ovarian cancer data, the distribution of absolute node score (T-statistic) is presented in the left-hand side and the distribution of node score terms in the objective function is presented in the right-hand side.

**Figure S5:** The Figure S5 shows that the standard simplex in two dimensional Euclidean space is the smallest of the four different norm subspaces and it is completely included in the other norm balls. Projection of any solution in the other spaces onto the standard simplex represents a more sparse density solution.

**Figure S6:** (A) If ***C*** is a convex set, then minimal distance from any point $\hat{x}$ onto ***C*** is unique and the projection $x^{*}$ can be uniquely found. (B) If ***C*** is a non-convex set, then minimal distance from any point $\hat{x}$ onto ***C*** is not unique. More than one solution can be found in general.

**Supplementary Tables**

**Table S1:** A list of 100 genes with the highest contribution scores to the condition specific network in the analysis of ovarian cancer data.

|  | Gene Symbol | Gene Entrez ID | Contribution score |
| --- | --- | --- | --- |
| 1 | CSF1R | 1436 | 0.002851987 |
| 2 | TNFRSF10A | 8797 | 0.002848172 |
| 3 | SELL | 6402 | 0.002848172 |
| 4 | HTR2A | 3356 | 0.002848172 |
| 5 | SMARCA4 | 6597 | 0.002844357 |
| 6 | PAX3 | 5077 | 0.002844357 |
| 7 | UBAP2L | 9898 | 0.002840542 |
| 8 | TFEB | 7942 | 0.002840542 |
| 9 | ITGB1 | 3688 | 0.002836728 |
| 10 | HES6 | 55502 | 0.002836728 |
| 11 | STAT5B | 6777 | 0.002832913 |
| 12 | BRAF | 673 | 0.002832913 |
| 13 | HSPA8 | 3312 | 0.002829098 |
| 14 | FIBP | 9158 | 0.002829098 |
| 15 | CDC73 | 79577 | 0.002829098 |
| 16 | STAT3 | 6774 | 0.002825284 |
| 17 | FGFR1OP | 11116 | 0.002825284 |
| 18 | PTN | 5764 | 0.002821469 |
| 19 | INSR | 3643 | 0.002821469 |
| 20 | DPPA4 | 55211 | 0.002821469 |
| 21 | RAD54B | 25788 | 0.002817654 |
| 22 | AANAT | 15 | 0.002817654 |
| 23 | SORBS1 | 10580 | 0.002813839 |
| 24 | NUB1 | 51667 | 0.002813839 |
| 25 | PLK4 | 10733 | 0.002810025 |
| 26 | CREBBP | 1387 | 0.002810025 |
| 27 | UPF3B | 65109 | 0.00280621 |
| 28 | RELA | 5970 | 0.00280621 |
| 29 | EGFR | 1956 | 0.00280621 |
| 30 | TP53 | 7157 | 0.002802395 |
| 31 | CAPNS1 | 826 | 0.002798581 |
| 32 | RECQL5 | 9400 | 0.002794766 |
| 33 | PKLR | 5313 | 0.002790951 |
| 34 | CMTM8 | 152189 | 0.002790951 |
| 35 | AKAP9 | 10142 | 0.002790951 |
| 36 | PPP2CA | 5515 | 0.002775692 |
| 37 | MSX2 | 4488 | 0.002775692 |
| 38 | SUB1 | 10923 | 0.002768063 |
| 39 | PICK1 | 9463 | 0.002760433 |
| 40 | AKT1S1 | 84335 | 0.002745175 |
| 41 | NONO | 4841 | 0.002737545 |
| 42 | TRIP13 | 9319 | 0.00273373 |
| 43 | CAMK2A | 815 | 0.002729916 |
| 44 | BCL2 | 596 | 0.002729916 |
| 45 | RAC1 | 5879 | 0.002726101 |
| 46 | SMAD3 | 4088 | 0.002722286 |
| 47 | GRB2 | 2885 | 0.002710842 |
| 48 | MAP3K5 | 4217 | 0.002699398 |
| 49 | HNRNPA2B1 | 3181 | 0.002699398 |
| 50 | SLC27A6 | 28965 | 0.002695583 |
| 51 | PRPS1 | 5631 | 0.002695583 |
| 52 | ZNF193 | 7746 | 0.002691769 |
| 53 | MEP1A | 4224 | 0.002691769 |
| 54 | LAMA4 | 3910 | 0.002691769 |
| 55 | PTPN2 | 5771 | 0.002684139 |
| 56 | GFI1B | 8328 | 0.002672695 |
| 57 | XPO1 | 7514 | 0.002665066 |
| 58 | UACA | 55075 | 0.002665066 |
| 59 | GHR | 2690 | 0.002665066 |
| 60 | CCDC85B | 11007 | 0.002661251 |
| 61 | TXN | 7295 | 0.002657436 |
| 62 | SUV39H1 | 6839 | 0.002653622 |
| 63 | GTF2A1 | 2957 | 0.002653622 |
| 64 | MAGI2 | 9863 | 0.002645992 |
| 65 | BRCA1 | 672 | 0.002645992 |
| 66 | ACTA1 | 58 | 0.002642177 |
| 67 | SVEP1 | 79987 | 0.002638363 |
| 68 | UBQLN4 | 56893 | 0.002626919 |
| 69 | ANKRD2 | 26287 | 0.002626919 |
| 70 | RB1 | 5925 | 0.002623104 |
| 71 | NRIP2 | 83714 | 0.002623104 |
| 72 | RCC1 | 1104 | 0.002615474 |
| 73 | MED31 | 51003 | 0.002615474 |
| 74 | CCR7 | 1236 | 0.002615474 |
| 75 | NIN | 51199 | 0.00261166 |
| 76 | DDIT3 | 1649 | 0.002607845 |
| 77 | TRAF1 | 7185 | 0.00260403 |
| 78 | FGD6 | 55785 | 0.00260403 |
| 79 | SCRIB | 23513 | 0.002592586 |
| 80 | PCSK6 | 5046 | 0.002592586 |
| 81 | EEF1A1 | 1915 | 0.002588771 |
| 82 | PPP2CB | 5516 | 0.002581142 |
| 83 | RIPK2 | 8767 | 0.002577327 |
| 84 | MMP1 | 4312 | 0.002573513 |
| 85 | SMURF1 | 57154 | 0.002569698 |
| 86 | STK39 | 27347 | 0.002565883 |
| 87 | KNG1 | 3827 | 0.002562068 |
| 88 | SLC9A3R1 | 9368 | 0.002542995 |
| 89 | MAP2K1 | 5604 | 0.00253918 |
| 90 | ELAC2 | 60528 | 0.00253918 |
| 91 | SRPK2 | 6733 | 0.002535365 |
| 92 | GTF2F1 | 2962 | 0.002527736 |
| 93 | GRAP2 | 9402 | 0.002527736 |
| 94 | DFNA5 | 1687 | 0.002527736 |
| 95 | KDR | 3791 | 0.002516292 |
| 96 | SNCG | 6623 | 0.002501033 |
| 97 | SOX9 | 6662 | 0.002497218 |
| 98 | UBTF | 7343 | 0.002493404 |
| 99 | PRKCD | 5580 | 0.002493404 |
| 100 | KLF6 | 1316 | 0.002489589 |

**Table S2:** A list of 100 genes with the highest contribution scores to the condition specific network in the analysis of prostate cancer data .

|  | **Gene Symbol** | **Gene Entrez ID** | **Contribution score** |
| --- | --- | --- | --- |
| 1 | C7orf36 | 57002 | 0.003526 |
| 2 | NID2 | 22795 | 0.003514 |
| 3 | GRB10 | 2887 | 0.003514 |
| 4 | CREBBP | 1387 | 0.003507 |
| 5 | MLLT4 | 4301 | 0.003503 |
| 6 | KCNK3 | 3777 | 0.003499 |
| 7 | GOLM1 | 51280 | 0.003491 |
| 8 | WIPI1 | 55062 | 0.003487 |
| 9 | PIK3R1 | 5295 | 0.003484 |
| 10 | RPS6KA1 | 6195 | 0.003457 |
| 11 | PRKAR1A | 5573 | 0.003457 |
| 12 | F8 | 2157 | 0.003442 |
| 13 | JUN | 3725 | 0.00343 |
| 14 | MXD3 | 83463 | 0.003423 |
| 15 | EP300 | 2033 | 0.003415 |
| 16 | EGFR | 1956 | 0.003415 |
| 17 | ADAM15 | 8751 | 0.003407 |
| 18 | DNMT1 | 1786 | 0.003404 |
| 19 | ANTXR2 | 118429 | 0.003404 |
| 20 | TTK | 7272 | 0.0034 |
| 21 | MST1R | 4486 | 0.0034 |
| 22 | CCDC130 | 81576 | 0.003373 |
| 23 | ATN1 | 1822 | 0.003365 |
| 24 | DAZAP2 | 9802 | 0.003362 |
| 25 | PAK1IP1 | 55003 | 0.003358 |
| 26 | LCK | 3932 | 0.003358 |
| 27 | INPP5D | 3635 | 0.003358 |
| 28 | HSPA1A | 3303 | 0.003331 |
| 29 | BPTF | 2186 | 0.003323 |
| 30 | CAMK2A | 815 | 0.003304 |
| 31 | ELAVL1 | 1994 | 0.003301 |
| 32 | EED | 8726 | 0.003285 |
| 33 | RAP2A | 5911 | 0.003278 |
| 34 | DSTN | 11034 | 0.003278 |
| 35 | EPHA4 | 2043 | 0.003274 |
| 36 | GATA2 | 2624 | 0.00327 |
| 37 | CBLB | 868 | 0.003266 |
| 38 | GJA1 | 2697 | 0.003262 |
| 39 | PRKCG | 5582 | 0.003262 |
| 40 | SIN3A | 25942 | 0.003259 |
| 41 | PROX1 | 5629 | 0.003259 |
| 42 | DCP1B | 196513 | 0.003259 |
| 43 | JUND | 3727 | 0.003255 |
| 44 | IKBKG | 8517 | 0.003255 |
| 45 | MAPK1 | 5594 | 0.003251 |
| 46 | SERPINE1 | 5054 | 0.003243 |
| 47 | NR1I2 | 8856 | 0.003236 |
| 48 | CD44 | 960 | 0.003236 |
| 49 | HABP4 | 22927 | 0.003224 |
| 50 | RCHY1 | 25898 | 0.003209 |
| 51 | OCLN | 4950 | 0.003205 |
| 52 | MAFK | 7975 | 0.003201 |
| 53 | YWHAE | 7531 | 0.003198 |
| 54 | NFKBIA | 4792 | 0.003186 |
| 55 | PCNA | 5111 | 0.003167 |
| 56 | SUMO1 | 7341 | 0.003163 |
| 57 | PRKD1 | 5587 | 0.003159 |
| 58 | ITGA4 | 3676 | 0.003159 |
| 59 | SMAD2 | 4087 | 0.003156 |
| 60 | HAND2 | 9464 | 0.003156 |
| 61 | MEF2A | 4205 | 0.003152 |
| 62 | BCL3 | 602 | 0.003144 |
| 63 | NR1I3 | 9970 | 0.00314 |
| 64 | HRAS | 3265 | 0.00314 |
| 65 | NOTCH2 | 4853 | 0.003133 |
| 66 | EXOC4 | 60412 | 0.003121 |
| 67 | MAP3K7IP1 | 10454 | 0.003114 |
| 68 | EPN1 | 29924 | 0.003114 |
| 69 | SYNCRIP | 10492 | 0.003098 |
| 70 | PRSS1 | 5644 | 0.003098 |
| 71 | AKAP9 | 10142 | 0.003095 |
| 72 | ATP2B4 | 493 | 0.003091 |
| 73 | RNF183 | 138065 | 0.003079 |
| 74 | PZP | 5858 | 0.003079 |
| 75 | MGMT | 4255 | 0.003068 |
| 76 | PRKG1 | 5592 | 0.003068 |
| 77 | ERBB4 | 2066 | 0.00306 |
| 78 | TRADD | 8717 | 0.003056 |
| 79 | WNT3 | 7473 | 0.003053 |
| 80 | CRMP1 | 1400 | 0.003053 |
| 81 | ZMYND8 | 23613 | 0.003049 |
| 82 | ILK | 3611 | 0.003026 |
| 83 | GEMIN4 | 50628 | 0.003026 |
| 84 | ABL1 | 25 | 0.003026 |
| 85 | AATF | 26574 | 0.003026 |
| 86 | MITF | 4286 | 0.002999 |
| 87 | ATP5J2 | 9551 | 0.002992 |
| 88 | ZNF274 | 10782 | 0.00298 |
| 89 | RPA1 | 6117 | 0.00298 |
| 90 | PLCG1 | 5335 | 0.00298 |
| 91 | NOTCH1 | 4851 | 0.002972 |
| 92 | TUBA4A | 7277 | 0.002969 |
| 93 | EZH2 | 2146 | 0.002969 |
| 94 | EEF1A1 | 1915 | 0.002965 |
| 95 | COL13A1 | 1305 | 0.002953 |
| 96 | KRT18 | 3875 | 0.002953 |
| 97 | GRB7 | 2886 | 0.00295 |
| 98 | MED31 | 51003 | 0.002946 |
| 99 | ATM | 472 | 0.002946 |
| 100 | LDLR | 3949 | 0.002938 |

**Table S3:** Performance on prostate cancer data.

| Method | COSINE | BMRF | WMAXC |
| --- | --- | --- | --- |
| Selected genes | 243 | 601 | 539 |
| Recovered interactions | 102 | 1179 | 1698 |
| Recovered genes | 23 | 94 | 95 |
| Fold enrichment | 1.262 | 2.086 | 2.35 |

Fold enrichment was used to evaluate the performance of methods, and calculated as follows:$Fold enrichment=\frac{{}^{'}Recoveredgenes^{'}*^{'}All genes'}{{}^{'}Selected genes^{'}*^{'}Referencegnes'}$ , where 'Selected genes' represents the number of selected genes by the method, 'Reference genes' is the number of reference genes from Prostate Cancer Dragon Database of genes, 'Recovered genes' is recovered genes by the method among reference genes, and 'All genes' represents all genes in the entire network. In the table, 'Recovered interactions' represents the number of interactions recovered from the PPI network.

**Table S4:** Literature evidences for 20 genes with the highest contribution scores to the condition specific network in the analysis of ovarian cancer data.

|  | Gene Symbol | Gene Entrez ID | Ovarian Cancer gene, reference | Gene, related to other, reference |
| --- | --- | --- | --- | --- |
| 1 | CSF1R | 1436 | [11] |  |
| 2 | TNFRSF10A | 8797 | [11] |  |
| 3 | SELL | 6402 |  | [12] |
| 4 | HTR2A | 3356 | [13] |  |
| 5 | SMARCA4 | 6597 | [14] |  |
| 6 | PAX3 | 5077 | [15] |  |
| 7 | UBAP2L | 9898 |  | [16] |
| 8 | TFEB | 7942 |  | [17] |
| 9 | ITGB1 | 3688 | [18] |  |
| 10 | HES6 | 55502 | [19] |  |
| 11 | STAT5B | 6777 | [20] |  |
| 12 | BRAF | 673 | [11] |  |
| 13 | HSPA8 | 3312 | [21] |  |
| 14 | FIBP | 9158 | [22] |  |
| 15 | CDC73 | 79577 | [23] |  |
| 16 | STAT3 | 6774 | [11] |  |
| 17 | FGFR1OP | 11116 | [24] |  |
| 18 | PTN | 5764 | [25] |  |
| 19 | INSR | 3643 | [11] |  |
| 20 | DPPA4 | 55211 |  | [26] |

**Table S5:** Neighbors of the four candidate ovarian cancer genes (UBAP2L,DPPA4,TFEB and SELL) are given in the table. For each neighbor gene, NCBI PubMed ID of the article supporting that the neighbor gene is related to ovarian cancer or another cancer type is given as a reference.

For the four candidate genes, 159 neighbors were found from the condition specific network consisting of 643 genes. Within the 159 neighbors, 94 were ovarian cancer-related genes, 57 were other cancer-related genes and 8 genes were unknown to be related to any cancer type.

|  | Gene Symbol | Ovarian cancer | Other cancers |
| --- | --- | --- | --- |
| 1 | AANAT | 10191081 |  |
| 2 | ADRA1D |  | 18817363 |
| 3 | AKR7A2 |  | 22040021 |
| 4 | AMBP |  | 24139944 |
| 5 | AQP1 | 24592091 |  |
| 6 | ASGR2 | unknown | |
| 7 | BAD | 18790805 |  |
| 8 | BAG1 | 18790805 |  |
| 9 | BAG3 | 21316839 |  |
| 10 | BCL2 | 18790805 |  |
| 11 | C1orf103 |  | 24270157 |
| 12 | CA4 | 18074350 |  |
| 13 | CAMK2G |  | 21649900 |
| 14 | CCNB1 | 24535252 |  |
| 15 | CD247 |  | 24244363 |
| 16 | CD4 | 24598551 |  |
| 17 | CD46 | 24210101 |  |
| 18 | CDKN1A | 24519527 |  |
| 19 | CLASP2 |  | 24522006 |
| 20 | CLSTN1 | 21118496 |  |
| 21 | CNOT7 |  | 23386060 |
| 22 | COPB1 | unknown | |
| 23 | CREBBP | 16732329 |  |
| 24 | CRKL | 21319228 |  |
| 25 | CRYAA |  | 21137063 |
| 26 | CYCS |  | 23770345 |
| 27 | DDIT3 | 23820265 |  |
| 28 | DFNA5 |  | 22530481 |
| 29 | DHFR | 24450514 |  |
| 30 | DHX9 | 12592385 |  |
| 31 | DLG1 |  | 24462519 |
| 32 | DNM2 |  | 23603912 |
| 33 | DPP4 | 22736146 |  |
| 34 | DSP | 1560038 |  |
| 35 | DYRK1B | 23528858 |  |
| 36 | EGFR | 18790805 |  |
| 37 | EIF3I |  | 24056964 |
| 38 | EIF4ENIF1 |  | 22397984 |
| 39 | FBLN5 | 21122382 |  |
| 40 | FHL1 | 22734036 |  |
| 41 | BRAF | 18790805 |  |
| 42 | FRS2 |  | 23393200 |
| 43 | GEMIN4 | 21636674 |  |
| 44 | GNAI1 | 21520077 |  |
| 45 | HAGH | 24605135 |  |
| 46 | HCK | 24145282 |  |
| 47 | HIST1H3A | 24603286 |  |
| 48 | HNF4A | 18844932 |  |
| 49 | HSPA1A | 22528050 |  |
| 50 | HTN3 | unknown | |
| 51 | ICAM1 | 23933413 |  |
| 52 | IFNAR1 | 22130162 |  |
| 53 | IGFBP3 | 18790805 |  |
| 54 | INHBA | 24302632 |  |
| 55 | INPP5A |  | 24358143 |
| 56 | JAK1 | 24605135 |  |
| 57 | JUN | 24591877 |  |
| 58 | KCNJ2 |  | 20195514 |
| 59 | KCNJ6 |  | 24392765 |
| 60 | KHDRBS1 |  | 24403051 |
| 61 | KIF23 |  | 24391143 |
| 62 | KPTN | unknown | |
| 63 | KRAS | 18790805 |  |
| 64 | KRT15 |  | 23869193 |
| 65 | LEF1 | 24030860 |  |
| 66 | LMX1A | 23270808 |  |
| 67 | LRP2 |  | 24462510 |
| 68 | LYN | 23383610 |  |
| 69 | MAGI3 |  | 24586183 |
| 70 | MAP3K5 | 18790805 |  |
| 71 | MAP4K1 |  | 21993544 |
| 72 | MAP4K4 | 16537454 |  |
| 73 | MBD3 |  | 24385926 |
| 74 | MCM2 | 22653344 |  |
| 75 | MEGF10 | 23580569 |  |
| 76 | MSH2 | 18790805 |  |
| 77 | MYBPC3 |  | 17652099 |
| 78 | MYOM1 |  | 23236287 |
| 79 | NECAP2 |  | 23792445 |
| 80 | NEDD4 | 20363515 |  |
| 81 | NLGN2 | unknown | |
| 82 | NOTCH4 | 23699385 |  |
| 83 | HSPA8 | 9990085 |  |
| 84 | NPAS2 |  | 24402410 |
| 85 | NPFF |  | 22375000 |
| 86 | NTSR1 |  | 24357116 |
| 87 | OAZ1 | 12888889 |  |
| 88 | PAFAH1B3 |  | 11285245 |
| 89 | PCSK5 |  | 24454770 |
| 90 | PDCD6 | 22369209 |  |
| 91 | PDGFB | 22993324 |  |
| 92 | PDGFRB | 23860180 |  |
| 93 | PICK1 | 20384629 |  |
| 94 | PIP5K1A |  | 23219993 |
| 95 | PLEKHM1 | 23535648 |  |
| 96 | PLK4 |  | 24389189 |
| 97 | PNRC1 | 11768609 |  |
| 98 | PPP2CA | 24472300 |  |
| 99 | PPP2R5E |  | 24448818 |
| 100 | PRB4 |  | 23587162 |
| 101 | PRKAB1 |  | 21957981 |
| 102 | PRMT2 |  | 24292672 |
| 103 | PSMB5 |  | 23934082 |
| 104 | PXN | 15739201 |  |
| 105 | RAB3B | 24477653 |  |
| 106 | RAD51 | 24599673 |  |
| 107 | RAD54B | 22752289 |  |
| 108 | RIPK2 |  | 24128261 |
| 109 | RPL31 |  | 22714919 |
| 110 | RPL5 |  | 24061479 |
| 111 | SCN5A | 20372843 |  |
| 112 | SCRIB | 22658591 |  |
| 113 | SETDB1 | 23034801 |  |
| 114 | SFRS12 |  | 19951526 |
| 115 | SHBG | 24386165 |  |
| 116 | SHC1 | 23178716 |  |
| 117 | SMAD3 | 24308608 |  |
| 118 | SMARCA4 | 24375037 |  |
| 119 | SNAP29 |  | 11444812 |
| 120 | SP1 | 24510775 |  |
| 121 | SPTAN1 | 16773180 |  |
| 122 | STAT3 | 18790805 |  |
| 123 | STAT5B | 21543928 |  |
| 124 | STK39 |  | 23073627 |
| 125 | STX3 | unknown | |
| 126 | SUB1 | 19553991 |  |
| 127 | SUV39H1 | 16638127 |  |
| 128 | SVEP1 |  | 23317273 |
| 129 | TAF4B | 24068106 |  |
| 130 | TAF8 |  | 21682879 |
| 131 | TCEB2 |  | 20680678 |
| 132 | TCERG1 | 15483748 |  |
| 133 | TCF4 | 22232518 |  |
| 134 | TENC1 |  | 22786655 |
| 135 | TGFBR1 | 22905183 |  |
| 136 | TICAM1 |  | 22994872 |
| 137 | TIFA |  | 12566447 |
| 138 | TLN1 | 23722670 |  |
| 139 | TLR4 | 24527095 |  |
| 140 | TNFSF8 | 9111512 |  |
| 141 | TNFSF9 | 21520164 |  |
| 142 | TNNI3 |  | 20124440 |
| 143 | TOPBP1 | 23819404 |  |
| 144 | TPT1 | 20705598 |  |
| 145 | TRIM24 |  | 22666376 |
| 146 | TSC1 | 24170201 |  |
| 147 | TTR | 18790805 |  |
| 148 | TXN | 21799032 |  |
| 149 | TYROBP | 16339517 |  |
| 150 | UBE2I | 21971700 |  |
| 151 | UNC119 | unknown | |
| 152 | VEGFA | 18790805 |  |
| 153 | VHL | 24549370 |  |
| 154 | VIM | 23880734 |  |
| 155 | WASL |  | 23874846 |
| 156 | WDR5 |  | 24279307 |
| 157 | WDR89 | unknown | |
| 158 | WTAP | 21881488 |  |
| 159 | XRCC1 | 18790805 |  |

**Table S6:** Enriched KEGG pathways in the sub-network identified using the WMAXC method for the ovarian cancer data set.

| [Term](http://david.abcc.ncifcrf.gov/chartReport.jsp?d-16544-s=2&d-16544-o=2&d-16544-p=1&annot=47) | [P-Value](http://david.abcc.ncifcrf.gov/chartReport.jsp?d-16544-s=7&d-16544-o=1&d-16544-p=1&annot=47) | [Benjamini](http://david.abcc.ncifcrf.gov/chartReport.jsp?d-16544-s=8&d-16544-o=1&d-16544-p=1&annot=47) |
| --- | --- | --- |
| [Neurotrophin signaling pathway](http://david.abcc.ncifcrf.gov/kegg.jsp?path=hsa04722$Neurotrophin%20signaling%20pathway&termId=470038877&source=kegg) | 4.6E-21 | 6.0E-19 |
| [Pathways in cancer](http://david.abcc.ncifcrf.gov/kegg.jsp?path=hsa05200$Pathways%20in%20cancer&termId=470038899&source=kegg) | 3.6E-20 | 2.4E-18 |
| [Cell cycle](http://david.abcc.ncifcrf.gov/kegg.jsp?path=hsa04110$Cell%20cycle&termId=470038834&source=kegg) | 6.3E-14 | 2.8E-12 |
| [Pancreatic cancer](http://david.abcc.ncifcrf.gov/kegg.jsp?path=hsa05212$Pancreatic%20cancer&termId=470038902&source=kegg) | 3.6E-13 | 1.2E-11 |
| [Chronic myeloid leukemia](http://david.abcc.ncifcrf.gov/kegg.jsp?path=hsa05220$Chronic%20myeloid%20leukemia&termId=470038910&source=kegg) | 1.0E-12 | 2.7E-11 |
| [Prostate cancer](http://david.abcc.ncifcrf.gov/kegg.jsp?path=hsa05215$Prostate%20cancer&termId=470038905&source=kegg) | 1.1E-11 | 2.4E-10 |
| [ErbB signaling pathway](http://david.abcc.ncifcrf.gov/kegg.jsp?path=hsa04012$ErbB%20signaling%20pathway&termId=470038828&source=kegg) | 4.2E-11 | 7.9E-10 |
| [Glioma](http://david.abcc.ncifcrf.gov/kegg.jsp?path=hsa05214$Glioma&termId=470038904&source=kegg) | 7.2E-11 | 1.2E-9 |
| [T cell receptor signaling pathway](http://david.abcc.ncifcrf.gov/kegg.jsp?path=hsa04660$T%20cell%20receptor%20signaling%20pathway&termId=470038869&source=kegg) | 1.2E-9 | 1.8E-8 |
| [Small cell lung cancer](http://david.abcc.ncifcrf.gov/kegg.jsp?path=hsa05222$Small%20cell%20lung%20cancer&termId=470038912&source=kegg) | 4.5E-9 | 5.9E-8 |
| [Colorectal cancer](http://david.abcc.ncifcrf.gov/kegg.jsp?path=hsa05210$Colorectal%20cancer&termId=470038900&source=kegg) | 4.5E-9 | 5.9E-8 |
| [Non-small cell lung cancer](http://david.abcc.ncifcrf.gov/kegg.jsp?path=hsa05223$Non-small%20cell%20lung%20cancer&termId=470038913&source=kegg) | 1.3E-8 | 1.6E-7 |
| [Pathogenic Escherichia coli infection](http://david.abcc.ncifcrf.gov/kegg.jsp?path=hsa05130$Pathogenic%20Escherichia%20coli%20infection&termId=470038898&source=kegg) | 3.3E-8 | 3.6E-7 |
| [Oocyte meiosis](http://david.abcc.ncifcrf.gov/kegg.jsp?path=hsa04114$Oocyte%20meiosis&termId=470038835&source=kegg) | 4.4E-8 | 4.4E-7 |
| [Endometrial cancer](http://david.abcc.ncifcrf.gov/kegg.jsp?path=hsa05213$Endometrial%20cancer&termId=470038903&source=kegg) | 5.2E-8 | 4.8E-7 |
| [Natural killer cell mediated cytotoxicity](http://david.abcc.ncifcrf.gov/kegg.jsp?path=hsa04650$Natural%20killer%20cell%20mediated%20cytotoxicity&termId=470038868&source=kegg) | 1.3E-7 | 1.1E-6 |
| [Adherens junction](http://david.abcc.ncifcrf.gov/kegg.jsp?path=hsa04520$Adherens%20junction&termId=470038856&source=kegg) | 1.5E-7 | 1.2E-6 |
| [Renal cell carcinoma](http://david.abcc.ncifcrf.gov/kegg.jsp?path=hsa05211$Renal%20cell%20carcinoma&termId=470038901&source=kegg) | 1.6E-7 | 1.2E-6 |
| [Acute myeloid leukemia](http://david.abcc.ncifcrf.gov/kegg.jsp?path=hsa05221$Acute%20myeloid%20leukemia&termId=470038911&source=kegg) | 2.8E-7 | 2.0E-6 |
| [Chemokine signaling pathway](http://david.abcc.ncifcrf.gov/kegg.jsp?path=hsa04062$Chemokine%20signaling%20pathway&termId=470038831&source=kegg) | 3.9E-7 | 2.7E-6 |
| [Insulin signaling pathway](http://david.abcc.ncifcrf.gov/kegg.jsp?path=hsa04910$Insulin%20signaling%20pathway&termId=470038882&source=kegg) | 2.4E-6 | 1.6E-5 |
| [TGF-beta signaling pathway](http://david.abcc.ncifcrf.gov/kegg.jsp?path=hsa04350$TGF-beta%20signaling%20pathway&termId=470038850&source=kegg) | 5.1E-6 | 3.1E-5 |
| [Thyroid cancer](http://david.abcc.ncifcrf.gov/kegg.jsp?path=hsa05216$Thyroid%20cancer&termId=470038906&source=kegg) | 5.6E-6 | 3.3E-5 |
| [Focal adhesion](http://david.abcc.ncifcrf.gov/kegg.jsp?path=hsa04510$Focal%20adhesion&termId=470038853&source=kegg) | 5.8E-6 | 3.3E-5 |
| [Tight junction](http://david.abcc.ncifcrf.gov/kegg.jsp?path=hsa04530$Tight%20junction&termId=470038857&source=kegg) | 7.3E-6 | 3.9E-5 |
| [B cell receptor signaling pathway](http://david.abcc.ncifcrf.gov/kegg.jsp?path=hsa04662$B%20cell%20receptor%20signaling%20pathway&termId=470038870&source=kegg) | 1.1E-5 | 5.9E-5 |
| [MAPK signaling pathway](http://david.abcc.ncifcrf.gov/kegg.jsp?path=hsa04010$MAPK%20signaling%20pathway&termId=470038827&source=kegg) | 1.9E-5 | 9.4E-5 |
| [Melanoma](http://david.abcc.ncifcrf.gov/kegg.jsp?path=hsa05218$Melanoma&termId=470038908&source=kegg) | 2.4E-5 | 1.2E-4 |
| [Bladder cancer](http://david.abcc.ncifcrf.gov/kegg.jsp?path=hsa05219$Bladder%20cancer&termId=470038909&source=kegg) | 3.7E-5 | 1.7E-4 |
| [Toll-like receptor signaling pathway](http://david.abcc.ncifcrf.gov/kegg.jsp?path=hsa04620$Toll-like%20receptor%20signaling%20pathway&termId=470038862&source=kegg) | 4.4E-5 | 2.0E-4 |
| [VEGF signaling pathway](http://david.abcc.ncifcrf.gov/kegg.jsp?path=hsa04370$VEGF%20signaling%20pathway&termId=470038852&source=kegg) | 4.8E-5 | 2.1E-4 |
| [Long-term potentiation](http://david.abcc.ncifcrf.gov/kegg.jsp?path=hsa04720$Long-term%20potentiation&termId=470038876&source=kegg) | 6.1E-5 | 2.6E-4 |
| [Fc epsilon RI signaling pathway](http://david.abcc.ncifcrf.gov/kegg.jsp?path=hsa04664$Fc%20epsilon%20RI%20signaling%20pathway&termId=470038871&source=kegg) | 7.7E-5 | 3.1E-4 |
| [Gap junction](http://david.abcc.ncifcrf.gov/kegg.jsp?path=hsa04540$Gap%20junction&termId=470038858&source=kegg) | 1.0E-4 | 4.1E-4 |
| [Type II diabetes mellitus](http://david.abcc.ncifcrf.gov/kegg.jsp?path=hsa04930$Type%20II%20diabetes%20mellitus&termId=470038887&source=kegg) | 1.1E-4 | 4.3E-4 |
| [Apoptosis](http://david.abcc.ncifcrf.gov/kegg.jsp?path=hsa04210$Apoptosis&termId=470038843&source=kegg) | 2.8E-4 | 1.0E-3 |
| [Long-term depression](http://david.abcc.ncifcrf.gov/kegg.jsp?path=hsa04730$Long-term%20depression&termId=470038878&source=kegg) | 2.9E-4 | 1.0E-3 |
| [GnRH signaling pathway](http://david.abcc.ncifcrf.gov/kegg.jsp?path=hsa04912$GnRH%20signaling%20pathway&termId=470038883&source=kegg) | 3.3E-4 | 1.2E-3 |
| [Leukocyte transendothelial migration](http://david.abcc.ncifcrf.gov/kegg.jsp?path=hsa04670$Leukocyte%20transendothelial%20migration&termId=470038873&source=kegg) | 3.4E-4 | 1.2E-3 |
| [Jak-STAT signaling pathway](http://david.abcc.ncifcrf.gov/kegg.jsp?path=hsa04630$Jak-STAT%20signaling%20pathway&termId=470038866&source=kegg) | 6.0E-4 | 2.0E-3 |
| [Dorso-ventral axis formation](http://david.abcc.ncifcrf.gov/kegg.jsp?path=hsa04320$Dorso-ventral%20axis%20formation&termId=470038847&source=kegg) | 6.4E-4 | 2.1E-3 |
| [Fc gamma R-mediated phagocytosis](http://david.abcc.ncifcrf.gov/kegg.jsp?path=hsa04666$Fc%20gamma%20R-mediated%20phagocytosis&termId=470038872&source=kegg) | 7.4E-4 | 2.3E-3 |
| [Progesterone-mediated oocyte maturation](http://david.abcc.ncifcrf.gov/kegg.jsp?path=hsa04914$Progesterone-mediated%20oocyte%20maturation&termId=470038884&source=kegg) | 8.1E-4 | 2.5E-3 |
| [Epithelial cell signaling in Helicobacter pylori infection](http://david.abcc.ncifcrf.gov/kegg.jsp?path=hsa05120$Epithelial%20cell%20signaling%20in%20Helicobacter%20pylori%20infection&termId=470038897&source=kegg) | 9.1E-4 | 2.7E-3 |
| [Endocytosis](http://david.abcc.ncifcrf.gov/kegg.jsp?path=hsa04144$Endocytosis&termId=470038841&source=kegg) | 1.0E-3 | 3.1E-3 |
| [Notch signaling pathway](http://david.abcc.ncifcrf.gov/kegg.jsp?path=hsa04330$Notch%20signaling%20pathway&termId=470038848&source=kegg) | 2.2E-3 | 6.3E-3 |
| [Wnt signaling pathway](http://david.abcc.ncifcrf.gov/kegg.jsp?path=hsa04310$Wnt%20signaling%20pathway&termId=470038846&source=kegg) | 2.6E-3 | 7.3E-3 |
| [Aldosterone-regulated sodium reabsorption](http://david.abcc.ncifcrf.gov/kegg.jsp?path=hsa04960$Aldosterone-regulated%20sodium%20reabsorption&termId=470038890&source=kegg) | 3.4E-3 | 9.3E-3 |
| [Regulation of actin cytoskeleton](http://david.abcc.ncifcrf.gov/kegg.jsp?path=hsa04810$Regulation%20of%20actin%20cytoskeleton&termId=470038881&source=kegg) | 3.8E-3 | 1.0E-2 |
| [Viral myocarditis](http://david.abcc.ncifcrf.gov/kegg.jsp?path=hsa05416$Viral%20myocarditis&termId=470038923&source=kegg) | 4.3E-3 | 1.1E-2 |
| [mTOR signaling pathway](http://david.abcc.ncifcrf.gov/kegg.jsp?path=hsa04150$mTOR%20signaling%20pathway&termId=470038842&source=kegg) | 4.5E-3 | 1.2E-2 |
| [Adipocytokine signaling pathway](http://david.abcc.ncifcrf.gov/kegg.jsp?path=hsa04920$Adipocytokine%20signaling%20pathway&termId=470038886&source=kegg) | 8.3E-3 | 2.1E-2 |
| [Melanogenesis](http://david.abcc.ncifcrf.gov/kegg.jsp?path=hsa04916$Melanogenesis&termId=470038885&source=kegg) | 8.4E-3 | 2.1E-2 |
| [p53 signaling pathway](http://david.abcc.ncifcrf.gov/kegg.jsp?path=hsa04115$p53%20signaling%20pathway&termId=470038836&source=kegg) | 9.2E-3 | 2.2E-2 |
| [Phosphatidylinositol signaling system](http://david.abcc.ncifcrf.gov/kegg.jsp?path=hsa04070$Phosphatidylinositol%20signaling%20system&termId=470038832&source=kegg) | 1.6E-2 | 3.9E-2 |
| [Amyotrophic lateral sclerosis (ALS)](http://david.abcc.ncifcrf.gov/kegg.jsp?path=hsa05014$Amyotrophic%20lateral%20sclerosis%20(ALS)&termId=470038893&source=kegg) | 1.6E-2 | 3.8E-2 |
| [Homologous recombination](http://david.abcc.ncifcrf.gov/kegg.jsp?path=hsa03440$Homologous%20recombination&termId=470038825&source=kegg) | 2.8E-2 | 6.4E-2 |
| [Vascular smooth muscle contraction](http://david.abcc.ncifcrf.gov/kegg.jsp?path=hsa04270$Vascular%20smooth%20muscle%20contraction&termId=470038845&source=kegg) | 4.7E-2 | 1.0E-1 |
| [RIG-I-like receptor signaling pathway](http://david.abcc.ncifcrf.gov/kegg.jsp?path=hsa04622$RIG-I-like%20receptor%20signaling%20pathway&termId=470038864&source=kegg) | 7.5E-2 | 1.6E-1 |
| [NOD-like receptor signaling pathway](http://david.abcc.ncifcrf.gov/kegg.jsp?path=hsa04621$NOD-like%20receptor%20signaling%20pathway&termId=470038863&source=kegg) | 9.1E-2 | 1.9E-1 |

|  |  |  |  |  |  |  |
| --- | --- | --- | --- | --- | --- | --- |

**Table S7:** Enriched KEGG pathways in the sub-network identified using the WMAXC method for the prostate cancer data set.

| [Term](http://david.abcc.ncifcrf.gov/chartReport.jsp?d-16544-s=2&d-16544-o=2&d-16544-p=1&annot=47) | [P-Value](http://david.abcc.ncifcrf.gov/chartReport.jsp?d-16544-s=7&d-16544-o=1&d-16544-p=1&annot=47) | [Benjamini](http://david.abcc.ncifcrf.gov/chartReport.jsp?d-16544-s=8&d-16544-o=1&d-16544-p=1&annot=47) |
| --- | --- | --- |
| [Pathways in cancer](http://david.abcc.ncifcrf.gov/kegg.jsp?path=hsa05200$Pathways%20in%20cancer&termId=470038899&source=kegg) | 2.8E-29 | 3.4E-27 |
| [Neurotrophin signaling pathway](http://david.abcc.ncifcrf.gov/kegg.jsp?path=hsa04722$Neurotrophin%20signaling%20pathway&termId=470038877&source=kegg) | 1.0E-20 | 6.3E-19 |
| [Chronic myeloid leukemia](http://david.abcc.ncifcrf.gov/kegg.jsp?path=hsa05220$Chronic%20myeloid%20leukemia&termId=470038910&source=kegg) | 3.1E-20 | 1.3E-18 |
| [Prostate cancer](http://david.abcc.ncifcrf.gov/kegg.jsp?path=hsa05215$Prostate%20cancer&termId=470038905&source=kegg) | 1.1E-14 | 3.4E-13 |
| [Colorectal cancer](http://david.abcc.ncifcrf.gov/kegg.jsp?path=hsa05210$Colorectal%20cancer&termId=470038900&source=kegg) | 1.7E-14 | 4.1E-13 |
| [Focal adhesion](http://david.abcc.ncifcrf.gov/kegg.jsp?path=hsa04510$Focal%20adhesion&termId=470038853&source=kegg) | 3.5E-13 | 7.0E-12 |
| [MAPK signaling pathway](http://david.abcc.ncifcrf.gov/kegg.jsp?path=hsa04010$MAPK%20signaling%20pathway&termId=470038827&source=kegg) | 2.3E-12 | 4.0E-11 |
| [ErbB signaling pathway](http://david.abcc.ncifcrf.gov/kegg.jsp?path=hsa04012$ErbB%20signaling%20pathway&termId=470038828&source=kegg) | 1.9E-11 | 3.0E-10 |
| [Apoptosis](http://david.abcc.ncifcrf.gov/kegg.jsp?path=hsa04210$Apoptosis&termId=470038843&source=kegg) | 1.3E-10 | 1.8E-9 |
| [B cell receptor signaling pathway](http://david.abcc.ncifcrf.gov/kegg.jsp?path=hsa04662$B%20cell%20receptor%20signaling%20pathway&termId=470038870&source=kegg) | 2.1E-10 | 2.6E-9 |
| [Glioma](http://david.abcc.ncifcrf.gov/kegg.jsp?path=hsa05214$Glioma&termId=470038904&source=kegg) | 3.0E-10 | 3.3E-9 |
| [Endometrial cancer](http://david.abcc.ncifcrf.gov/kegg.jsp?path=hsa05213$Endometrial%20cancer&termId=470038903&source=kegg) | 4.8E-10 | 4.9E-9 |
| [Small cell lung cancer](http://david.abcc.ncifcrf.gov/kegg.jsp?path=hsa05222$Small%20cell%20lung%20cancer&termId=470038912&source=kegg) | 2.3E-9 | 2.2E-8 |
| [Adherens junction](http://david.abcc.ncifcrf.gov/kegg.jsp?path=hsa04520$Adherens%20junction&termId=470038856&source=kegg) | 2.5E-9 | 2.1E-8 |
| [T cell receptor signaling pathway](http://david.abcc.ncifcrf.gov/kegg.jsp?path=hsa04660$T%20cell%20receptor%20signaling%20pathway&termId=470038869&source=kegg) | 3.0E-9 | 2.5E-8 |
| [Cell cycle](http://david.abcc.ncifcrf.gov/kegg.jsp?path=hsa04110$Cell%20cycle&termId=470038834&source=kegg) | 3.5E-9 | 2.7E-8 |
| [Acute myeloid leukemia](http://david.abcc.ncifcrf.gov/kegg.jsp?path=hsa05221$Acute%20myeloid%20leukemia&termId=470038911&source=kegg) | 3.6E-9 | 2.6E-8 |
| [Pancreatic cancer](http://david.abcc.ncifcrf.gov/kegg.jsp?path=hsa05212$Pancreatic%20cancer&termId=470038902&source=kegg) | 4.2E-9 | 2.8E-8 |
| [Non-small cell lung cancer](http://david.abcc.ncifcrf.gov/kegg.jsp?path=hsa05223$Non-small%20cell%20lung%20cancer&termId=470038913&source=kegg) | 7.8E-9 | 5.0E-8 |
| [Wnt signaling pathway](http://david.abcc.ncifcrf.gov/kegg.jsp?path=hsa04310$Wnt%20signaling%20pathway&termId=470038846&source=kegg) | 1.6E-8 | 9.6E-8 |
| [TGF-beta signaling pathway](http://david.abcc.ncifcrf.gov/kegg.jsp?path=hsa04350$TGF-beta%20signaling%20pathway&termId=470038850&source=kegg) | 2.7E-8 | 1.5E-7 |
| [Renal cell carcinoma](http://david.abcc.ncifcrf.gov/kegg.jsp?path=hsa05211$Renal%20cell%20carcinoma&termId=470038901&source=kegg) | 9.5E-8 | 5.3E-7 |
| [Fc epsilon RI signaling pathway](http://david.abcc.ncifcrf.gov/kegg.jsp?path=hsa04664$Fc%20epsilon%20RI%20signaling%20pathway&termId=470038871&source=kegg) | 5.6E-7 | 3.0E-6 |
| [Insulin signaling pathway](http://david.abcc.ncifcrf.gov/kegg.jsp?path=hsa04910$Insulin%20signaling%20pathway&termId=470038882&source=kegg) | 1.3E-6 | 6.5E-6 |
| [Endocytosis](http://david.abcc.ncifcrf.gov/kegg.jsp?path=hsa04144$Endocytosis&termId=470038841&source=kegg) | 1.3E-6 | 6.5E-6 |
| [Leukocyte transendothelial migration](http://david.abcc.ncifcrf.gov/kegg.jsp?path=hsa04670$Leukocyte%20transendothelial%20migration&termId=470038873&source=kegg) | 1.6E-6 | 7.4E-6 |
| [Long-term potentiation](http://david.abcc.ncifcrf.gov/kegg.jsp?path=hsa04720$Long-term%20potentiation&termId=470038876&source=kegg) | 1.8E-6 | 8.3E-6 |
| [Chemokine signaling pathway](http://david.abcc.ncifcrf.gov/kegg.jsp?path=hsa04062$Chemokine%20signaling%20pathway&termId=470038831&source=kegg) | 1.9E-6 | 8.2E-6 |
| [Gap junction](http://david.abcc.ncifcrf.gov/kegg.jsp?path=hsa04540$Gap%20junction&termId=470038858&source=kegg) | 4.3E-6 | 1.8E-5 |
| [Melanoma](http://david.abcc.ncifcrf.gov/kegg.jsp?path=hsa05218$Melanoma&termId=470038908&source=kegg) | 1.6E-5 | 6.5E-5 |
| [GnRH signaling pathway](http://david.abcc.ncifcrf.gov/kegg.jsp?path=hsa04912$GnRH%20signaling%20pathway&termId=470038883&source=kegg) | 1.8E-5 | 7.0E-5 |
| [Melanogenesis](http://david.abcc.ncifcrf.gov/kegg.jsp?path=hsa04916$Melanogenesis&termId=470038885&source=kegg) | 2.1E-5 | 7.8E-5 |
| [Pathogenic Escherichia coli infection](http://david.abcc.ncifcrf.gov/kegg.jsp?path=hsa05130$Pathogenic%20Escherichia%20coli%20infection&termId=470038898&source=kegg) | 2.5E-5 | 9.2E-5 |
| [Regulation of actin cytoskeleton](http://david.abcc.ncifcrf.gov/kegg.jsp?path=hsa04810$Regulation%20of%20actin%20cytoskeleton&termId=470038881&source=kegg) | 3.1E-5 | 1.1E-4 |
| [Natural killer cell mediated cytotoxicity](http://david.abcc.ncifcrf.gov/kegg.jsp?path=hsa04650$Natural%20killer%20cell%20mediated%20cytotoxicity&termId=470038868&source=kegg) | 4.0E-5 | 1.4E-4 |
| [Epithelial cell signaling in Helicobacter pylori infection](http://david.abcc.ncifcrf.gov/kegg.jsp?path=hsa05120$Epithelial%20cell%20signaling%20in%20Helicobacter%20pylori%20infection&termId=470038897&source=kegg) | 4.2E-5 | 1.4E-4 |
| [Notch signaling pathway](http://david.abcc.ncifcrf.gov/kegg.jsp?path=hsa04330$Notch%20signaling%20pathway&termId=470038848&source=kegg) | 8.2E-5 | 2.7E-4 |
| [Tight junction](http://david.abcc.ncifcrf.gov/kegg.jsp?path=hsa04530$Tight%20junction&termId=470038857&source=kegg) | 1.4E-4 | 4.4E-4 |
| [Bladder cancer](http://david.abcc.ncifcrf.gov/kegg.jsp?path=hsa05219$Bladder%20cancer&termId=470038909&source=kegg) | 1.5E-4 | 4.6E-4 |
| [Adipocytokine signaling pathway](http://david.abcc.ncifcrf.gov/kegg.jsp?path=hsa04920$Adipocytokine%20signaling%20pathway&termId=470038886&source=kegg) | 1.5E-4 | 4.5E-4 |
| [Long-term depression](http://david.abcc.ncifcrf.gov/kegg.jsp?path=hsa04730$Long-term%20depression&termId=470038878&source=kegg) | 2.0E-4 | 6.0E-4 |
| [Thyroid cancer](http://david.abcc.ncifcrf.gov/kegg.jsp?path=hsa05216$Thyroid%20cancer&termId=470038906&source=kegg) | 2.3E-4 | 6.7E-4 |
| [Toll-like receptor signaling pathway](http://david.abcc.ncifcrf.gov/kegg.jsp?path=hsa04620$Toll-like%20receptor%20signaling%20pathway&termId=470038862&source=kegg) | 3.2E-4 | 9.1E-4 |
| [VEGF signaling pathway](http://david.abcc.ncifcrf.gov/kegg.jsp?path=hsa04370$VEGF%20signaling%20pathway&termId=470038852&source=kegg) | 4.8E-4 | 1.3E-3 |
| [Fc gamma R-mediated phagocytosis](http://david.abcc.ncifcrf.gov/kegg.jsp?path=hsa04666$Fc%20gamma%20R-mediated%20phagocytosis&termId=470038872&source=kegg) | 5.1E-4 | 1.4E-3 |
| [Aldosterone-regulated sodium reabsorption](http://david.abcc.ncifcrf.gov/kegg.jsp?path=hsa04960$Aldosterone-regulated%20sodium%20reabsorption&termId=470038890&source=kegg) | 6.1E-4 | 1.6E-3 |
| [Oocyte meiosis](http://david.abcc.ncifcrf.gov/kegg.jsp?path=hsa04114$Oocyte%20meiosis&termId=470038835&source=kegg) | 8.5E-4 | 2.2E-3 |
| [Vascular smooth muscle contraction](http://david.abcc.ncifcrf.gov/kegg.jsp?path=hsa04270$Vascular%20smooth%20muscle%20contraction&termId=470038845&source=kegg) | 1.0E-3 | 2.6E-3 |
| [NOD-like receptor signaling pathway](http://david.abcc.ncifcrf.gov/kegg.jsp?path=hsa04621$NOD-like%20receptor%20signaling%20pathway&termId=470038863&source=kegg) | 1.1E-3 | 2.6E-3 |
| [p53 signaling pathway](http://david.abcc.ncifcrf.gov/kegg.jsp?path=hsa04115$p53%20signaling%20pathway&termId=470038836&source=kegg) | 2.3E-3 | 5.6E-3 |
| [RIG-I-like receptor signaling pathway](http://david.abcc.ncifcrf.gov/kegg.jsp?path=hsa04622$RIG-I-like%20receptor%20signaling%20pathway&termId=470038864&source=kegg) | 3.3E-3 | 7.8E-3 |
| [Amyotrophic lateral sclerosis (ALS)](http://david.abcc.ncifcrf.gov/kegg.jsp?path=hsa05014$Amyotrophic%20lateral%20sclerosis%20(ALS)&termId=470038893&source=kegg) | 4.1E-3 | 9.5E-3 |
| [Type II diabetes mellitus](http://david.abcc.ncifcrf.gov/kegg.jsp?path=hsa04930$Type%20II%20diabetes%20mellitus&termId=470038887&source=kegg) | 6.5E-3 | 1.5E-2 |
| [Dorso-ventral axis formation](http://david.abcc.ncifcrf.gov/kegg.jsp?path=hsa04320$Dorso-ventral%20axis%20formation&termId=470038847&source=kegg) | 1.5E-2 | 3.4E-2 |
| [Arrhythmogenic right ventricular cardiomyopathy (ARVC)](http://david.abcc.ncifcrf.gov/kegg.jsp?path=hsa05412$Arrhythmogenic%20right%20ventricular%20cardiomyopathy%20(ARVC)&termId=470038921&source=kegg) | 1.5E-2 | 3.4E-2 |
| [Primary immunodeficiency](http://david.abcc.ncifcrf.gov/kegg.jsp?path=hsa05340$Primary%20immunodeficiency&termId=470038919&source=kegg) | 1.7E-2 | 3.7E-2 |
| [Ubiquitin mediated proteolysis](http://david.abcc.ncifcrf.gov/kegg.jsp?path=hsa04120$Ubiquitin%20mediated%20proteolysis&termId=470038837&source=kegg) | 1.8E-2 | 3.8E-2 |
| [Progesterone-mediated oocyte maturation](http://david.abcc.ncifcrf.gov/kegg.jsp?path=hsa04914$Progesterone-mediated%20oocyte%20maturation&termId=470038884&source=kegg) | 3.4E-2 | 7.0E-2 |
| [Calcium signaling pathway](http://david.abcc.ncifcrf.gov/kegg.jsp?path=hsa04020$Calcium%20signaling%20pathway&termId=470038829&source=kegg) | 3.7E-2 | 7.4E-2 |
| [Basal cell carcinoma](http://david.abcc.ncifcrf.gov/kegg.jsp?path=hsa05217$Basal%20cell%20carcinoma&termId=470038907&source=kegg) | 4.7E-2 | 9.2E-2 |
| [Jak-STAT signaling pathway](http://david.abcc.ncifcrf.gov/kegg.jsp?path=hsa04630$Jak-STAT%20signaling%20pathway&termId=470038866&source=kegg) | 4.7E-2 | 9.2E-2 |
| [Axon guidance](http://david.abcc.ncifcrf.gov/kegg.jsp?path=hsa04360$Axon%20guidance&termId=470038851&source=kegg) | 9.0E-2 | 1.7E-1 |
